# Climatology and dynamics of the link between dry intrusions and cold fronts during winter. Part I: global climatology

**DOI:** 10.1007/s00382-019-04745-w

**Published:** 2019-05-07

**Authors:** Jennifer L. Catto, Shira Raveh-Rubin

**Affiliations:** 1 College of Engineering, Mathematics and Physical Sciences, University of Exeter, Exeter, UK; 2 Department of Earth and Planetary Sciences, Weizmann Institute of Science, Rehovot, Israel

**Keywords:** Atmospheric fronts, Dry intrusions, Extratropical cyclones, Trajectories, Climatology, Reanalysis

## Abstract

Cold fronts are a primary feature of the day-to-day variability of weather in the midlatitudes, and feature in conceptual extratropical cyclone models alongside the dry intrusion airstream. Here the climatological frequency and spatial distribution of the co-occurrence of these two features are quantified, and the differences in cold front characteristics (intensity, size, and precipitation) when a dry intrusion is present or not are calculated. Fronts are objectively identified in the ECMWF ERA-Interim dataset for the winter seasons in each hemisphere and split into three sub-types: central fronts (within a cyclone area); trailing fronts (outwith the cyclone area but connected to a central front); and isolated fronts (not connected to a cyclone). These are then associated with dry intrusions identified using Lagrangian trajectory analysis. Trailing fronts are most likely to be associated with a DI in both hemispheres, and this occurs more frequently in the western parts of the major storm track regions. Isolated fronts are linked to DIs more frequently on the eastern ends of the storm tracks, and in the subtropics. All front types, when co-occurring with a DI, are stronger in terms of their temperature gradient, are much larger in area, and typically have higher average precipitation. Therefore, climatologically the link with DIs increases the impact of cold fronts. There are some differences in the statistics of the precipitation for trailing and isolated fronts that are further investigated in Part II of this study from the front-centred perspective.

**Electronic supplementary material:**

The online version of this article (10.1007/s00382-019-04745-w) contains supplementary material, which is available to authorized users.

## Introduction

Extratropical cyclones and their associated warm and cold fronts are primary features for controlling the variability of weather in the midlatitudes. Dry intrusions (DIs) often accompany these extratropical cyclones and fronts, but their mutual occurrence has not been studied systematically. The goal of this two-part study is to better understand the connection between cold fronts and DIs, how this varies geographically, and what the joint characteristics are of the features themselves, their broader environment, and their impacts.

Fronts are typically defined as regions of strong temperature gradients and wind changes and lie at the interface of two air-masses of differing origin. They intensify as part of the secondary circulation within extratropical cyclones, with the frontogenesis dependent upon convergence and stretching and shearing deformation (Keyser et al. 1988). Fronts—particularly their development and impacts—have received a lot of attention in the way of case studies, theory, and numerical modeling (e.g., Browning 1986; Hoskins and Bretherton 1972; Sinclair and Keyser 2015).

Berry et al. (2011) was the first study to produce an automated global climatology of fronts, by applying the front identification concepts of Hewson (1998) to the European Centre for Medium Range Weather Forecasts (ECMWF) 40-year reanalysis dataset (ERA-40; Uppala et al. 2006). This method uses a thermal front parameter based on wet bulb potential temperature in order to identify frontal points, and then joins these points into linear features automatically. At the same time Simmonds et al. (2011) used a wind-shift method to identify fronts in the Southern Hemisphere (SH). There have been a number of other studies since then investigating fronts from a climatological perspective (e.g., Sinclair 2013; Solman and Orlanski 2014; Rudeva and Simmonds 2015; Utsumi et al. 2014; Schemm et al. 2015; Parfitt et al. 2017a; Spensberger and Sprenger 2018).

Such front climatologies show that in the Northern Hemisphere (NH) there are maximum frequencies in the winter, with the maxima following the major storm tracks in a southwest to northeast pattern across the North Atlantic and North Pacific Oceans (Berry et al. 2011; Parfitt et al. 2017a; Spensberger and Sprenger 2018). Cold fronts tend to occur more frequently in winter than warm fronts, and lie slightly further equatorward than the warm fronts as expected from the typical structure of extratropical cyclones.

In the SH winter, fronts are also identified with high frequency over the main storm tracks (Berry et al. 2011), across the South Atlantic, the Southern Indian Ocean, south of Australia and New Zealand, spiralling towards Antarctica (cf. Hoskins and Hodges 2005). As in the NH, the cold fronts are identified further equatorward than the warm fronts. These are features that are identified using thermal parameters as in Berry et al. (2011), the F diagnostic, using both temperature gradient and relative vorticity (Parfitt et al. 2017a), or the wind shift method of Simmonds et al. (2011). In some winter front climatologies there is also a clear maximum in front frequency in the South Pacific between $10^{\circ }\hbox {S}$ and $45^{\circ }\hbox {S}$ (Berry et al. 2011; Schemm et al. 2015; Spensberger and Sprenger 2018; Parfitt et al. 2017a), which consists of both warm and cold fronts (Berry et al. 2011).

There are some differences in the exact frequencies and the patterns depending on the front identification method used (e.g., Schemm et al. 2015; Thomas and Schultz 2018a, b). For example, Parfitt et al. (2017a) compared a front diagnostic using both temperature gradient and relative vorticity at 600 hPa (F diagnostic), with a thermal front parameter diagnostic similar to Berry et al. (2011) also on 600 hPa (T diagnostic), and found that in the western ocean basins, relatively more fronts were identified with the F than the T diagnostic, whereas in the eastern ocean basins, the opposite is true. This may reflect the lifecycles of the extratropical cyclones and the changing characteristics of fronts across the ocean basins. In this study we focus on lower level fronts, calculated using a thermal front parameter at 850 hPa. In general, the maximum front frequencies in the storm track regions are up to between 15 and 30%.

Fronts have certain associated characteristics that can be investigated using automated identification methods. Considering all frontal gridpoints, Catto et al. (2014) found that front intensity (defined as the gradient of wet bulb potential temperature across the front) is highest over the strong sea surface temperature gradient regions of the Gulf Stream and Kuroshio Current in the NH, and elsewhere generally increases with latitude. Simmonds et al. (2011), whose wind-shift detection algorithm is most suited to cold fronts, identified that the strongest fronts (defined in terms of their wind-shift) and the longest fronts, exist in the Southern Indian Ocean region. In this study we will address regional patterns of front frequency, as well as statistics on their intensity and size.

Conceptual understanding of the airflows within extratropical cyclones (Carlson 1980; Browning 1997) led to the definition of the warm conveyor belt (WCB), the cold conveyor belt and the DI. DIs are streams of air from the upper troposphere or lower stratosphere that descend towards the surface with low values of wet bulb potential temperature. These typically descend behind a cold front and fan out near the surface, with some of the airstream then ascending into the cloud head and some turning anticyclonically. The anomalously dry air can be seen in satellite imagery as the characteristic “dry-slot” (Browning 1997) behind a cold front.

Raveh-Rubin (2017) produced a climatology of DIs using Lagrangian trajectory analysis to identify streams of air that descend more than 400 hPa in 48 h. They found that DIs occur most commonly during the winter season in each hemisphere, with hardly any occurring during summer. Evaluation of the characteristics of the DIs for different regions revealed that there are three distinct groups of DIs—storm track DIs that occur over the North Pacific, North Atlantic and South Pacific; non-storm track DIs occurring in the Mediterranean, to the West of the US, and in the other Southern Ocean regions; and Antarctic DIs. The storm track DIs account for the highest frequency, particularly in the NH, consistent with the conceptual picture of DIs as part of the lifecycle of extratropical cyclones (Browning 1997). We would thus expect a high proportion of the DIs to be associated with frontal systems, and the frontal systems with DIs.

Both fronts and DIs are associated with distinct surface weather. The interaction of dry air within the DI with moister air near the surface ahead of the cold front can generate potential instability and give rise to convection (Browning 1997). DIs are known to interact with cold fronts in particular in different ways. If they undercut the moist air ahead of the cold front in the WCB, they can potentially suppress rainfall within the WCB by evaporation of raindrops falling into the dry airmass, but also force lifting of the moist airmass ahead of it (Raveh-Rubin and Wernli 2016). They may also overrun the moist air and cause convection to occur when potential instability is released (Browning and Golding 1995; Raveh-Rubin 2017). This can mean that DIs are also important for extreme weather events at the surface, such as severe winds (Browning and Reynolds 1994; Raveh-Rubin and Wernli 2015), or heavy precipitation (Browning and Golding 1995; Raveh-Rubin and Wernli 2016). By linking objectively identified fronts with estimates of precipitation, it has been shown that fronts are strongly associated with total and extreme precipitation in the midlatitudes (Catto et al. 2012; Catto and Pfahl 2013; Dowdy and Catto 2017; Utsumi et al. 2017). Catto and Pfahl (2013) showed that the fronts that produced the extreme precipitation events were up to 30% stronger in terms of their temperature gradients than the fronts that produced any type of precipitation, and that the extreme events could often be associated with cold fronts, including those trailing into the subtropics. Both warm fronts and cold fronts are frequently associated with WCBs (Catto et al. 2015), and this connection increases the likelihood of a front producing an extreme precipitation event. Fronts are also strongly associated with strong wind events either within the parent cyclone or along the trailing fronts in the warm conveyor belt region (Hewson and Neu 2015; Dowdy and Catto 2017). Although the frequency of occurrence of fronts and DIs is quite low, their association with a large proportion of extreme events indicates their importance and the need to further understand them.

Here we are interested in cold fronts in particular because of the structure of extratropical cyclones seen in conceptual models, and so we have not included an analysis of the link between DIs and warm fronts as we expect this to be much weaker. An interesting question is how often the co-occurrence of DIs and cold fronts really occurs and whether this has some impact on the strength, size, and impacts of the fronts. Do DIs always descend behind a cold front? Do cold fronts always occur with a DI? What is the impact on the cold sector of the extratropical cyclone (e.g., Vannière et al. 2016)? Since DIs are known to cause either the initiation or suppression of precipitation, it is an open question as to how the link between DIs and fronts will impact the frontal precipitation. Our goal is to develop a climatology of the link between cold fronts and DIs as a tool to guide our physical understanding of these features, and we systematically investigate the climatological impact of the association with DIs on the frontal precipitation and other frontal characteristics.

The particular questions part 1 of this two-part paper aims to answer are:

1. How often are DIs and cold fronts linked and what is the global spatial distribution of this linking?
2. Do the fronts that are linked to DIs have different characteristics compared to other fronts?

The impact of the DIs on the cold sector of extratropical cyclones has been documented, and in part 2 of this paper, will be analyzed from a front centered composite perspective to compare the cold sectors and front environments of cold fronts matched and not matched with DIs, and the spatial distribution of precipitation in each case.

The rest of this paper is laid out as follows. Section 2 gives an overview of the data used in the study, as well as the objective identification methods used to define fronts and DIs. This includes a detailed description of the technique used to separate the cold fronts into different classes, using an illustrative case. Section 3 gives the results of the global analysis of matching between cold fronts and DIs, as well as statistics of various front characteristics in the case of the fronts being matched or not-matched with DIs. A summary and discussion are given in Sect. 4.

## Data and methods

### Reanalysis data

The data used in this study come from the European Centre for Medium-Range Weather Forecasts (ECMWF) reanalysis product, ERA-Interim (Dee et al. 2011). The 6-hourly data from years 1979–2014 are used and are interpolated onto a $1^{\circ }$ by $1^{\circ }$ grid. Precipitation data from ERA-Interim are calculated from the Integrated Forecasting System (IFS) forecasts started from 00 UTC and 12 UTC. Therefore the precipitation amounts in our analysis are accumulated over the previous 6 h using the 6–12-h and 12–18-h lead time forecast accumulations. Since DIs occur most frequency during the winter season (Raveh-Rubin 2017), we focus on the seasons December to February (DJF) for the NH, and June to August (JJA) in the SH.

### Dry intrusion identification

The DIs are identified exactly as described in Raveh-Rubin (2017) using the Lagrangian analysis tool (LAGRANTO), version 2.0 (Sprenger and Wernli 2015) and ERA-Interim data at 6-hourly, $1^{\circ }$ horizontal resolution and 60 vertical hybrid levels. Forward trajectories are calculated from all points at altitudes higher than 600 hPa using the ERA-Interim wind fields starting from initial positions on a uniform grid with 80 km grid spacing and 20 hPa vertical spacing. Trajectories that increase their pressure (i.e. descend) by at least 400 hPa in 48 h are selected as DIs. This threshold value of descent was tested by Raveh-Rubin (2017) and was chosen to give the best representation of DIs. For a discussion of the sensitivity to DI definition criteria, readers are directed to the study of Raveh-Rubin (2017). The DI trajectories are converted into 2-dimensional Eulerian objects by projecting the trajectories at low levels (below 700 hPa) onto a $1^{\circ }$ grid. The number of trajectories within a grid box at each time is recorded and the DI objects are defined where there are more than $1\times10^{-5}$ trajectories per square km, using a contouring function.

### Cyclone identification

The cyclone identification method of Wernli and Schwierz (2006), recently used in Pfahl and Wernli (2012), Catto and Pfahl (2013), and Dowdy and Catto (2017), has been applied to the $1^{\circ }$ resolution ERA-Interim data. Cyclones are found by identifying closed contours of mean sea level pressure (MSLP) with contour intervals of 0.5 hPa. Regions where the topography is above 1500 m are excluded, and the outermost closed contour of a cyclone must be at least 100 km long. A major benefit of using this particular cyclone identification method (acknowledging that there are many different methods available, e.g. see Neu et al. (2013)), is that regions within the outermost closed pressure contour can be defined as the cyclone area. This has the advantage that fronts, DIs, and surface weather can be identified as being within or outside of the cyclone area without having to make any assumptions about cyclone size.

### Front identification

The fronts are identified using the method of Berry et al. (2011), following the work of Hewson (1998) using a thermal front parameter (*TFP*) based on gradients of wet bulb potential temperature ($\theta _{w}$) at 850 hPa. There has been much discussion in the literature about the appropriate thermal variable to use for the detection of fronts (Hewson 1998; Schemm et al. 2018; Thomas and Schultz 2018b), and while ($\theta _{w}$) may be considered to have a disadvantage in its sensitivity to moisture gradients (Sanders 1999), this may actually be a benefit when considering the connection of fronts to DIs, as DIs carry low $\theta _{w}$ due to both the potential temperature and the low moisture content. This quantity is also conserved for moist adiabatic processes and successfully detects fronts at all times of day. See Schemm et al. (2018) for an in depth discussion regarding front identification variables. Frontal points are identified where the gradient of *TFP* is zero, where $TFP(\theta _{w})=-\nabla |\nabla \theta _{w}|.(\nabla \theta _{w}/|\nabla \theta _{w}|)$, after the *TFP* field has been masked out above a threshold value (here we have used the same threshold as that used in Dowdy and Catto (2017)). In this study the fronts have been separated into warm and cold fronts depending on the advection of the contours of $\theta _{w}$ in the direction of the cold temperatures (the front speed). When the front speed is positive a warm front is defined, and where the front speed is negative a cold front is defined, as in Hewson (1998). The frontal points are joined using a line-joining algorithm, requiring the frontal points to be within a radius of $3^{\circ }$ of each other.

The fronts have been identified on the $0.75^{\circ }$ gridded data from ERA-Interim and regridded onto a $1^{\circ }$ grid to be consistent with the cyclones and DIs. We also impose a final requirement that the fronts must contain at least 5 grid boxes. At each time each front is given a unique identifier, and all grid points through which the front line passes are allocated with the same identifier value. This allows for easier separation into different front types (see next section) and easier matching with DIs.

Any automated feature identification method requires some parameter choices. For example, advantages and disadvantages of various methods of identifying fronts (including the thermal variable, the level, and the function of the thermal variable) can be found in Thomas and Schultz (2018a, b). We have examined the sensitivity of some of the results of the study for a single season to some of these parameter choices. For example, we have tested the impact of the maximum search radius for the line-joining, and the requirement of the minimum front size. We have also investigated the impact of identifying fronts on two different pressure levels (925 hPa and 700 hPa), and using a doubled and a halved *TFP* threshold. The results of these sensitivity tests can be seen in the supplementary material. While we find no change to the conclusions of the study based on these sensitivity tests, some of the features will be discussed in Sect. 4.

### Defining front object types

In order to better understand how DIs and cold fronts link, we have defined three types of cold fronts; central fronts, which are cold fronts (or sections of cold fronts) lying within the defined cyclone areas; trailing fronts, which are the sections of cold fronts lying outside of the defined cyclone areas but that have some part of their length defined as a central front; and isolated fronts, which are cold fronts fully outside of any cyclone area. This choice was made based on a number of factors. Inspection of a number of cases of DIs in Raveh-Rubin (2017) indicates that DIs often have an equatorward movement over their lifetime and would therefore likely be associated with fronts at lower latitudes. Cyclones themselves have been shown to be important for precipitation, with composites of cyclones indicating very high precipitation near the center of the cyclones (e.g. Hawcroft et al. 2017; Naud 2018). However, by using the same cyclone masks as we use here, Catto and Pfahl (2013) showed that much of the extreme precipitation not associated with cyclones is associated with trailing fronts. Our interest is in whether the DIs might have some influence on fronts that lie further away from the central part of the cyclone, and whether there is a difference depending on if the front is or is not associated with a midlatitude cyclone.

Since fronts have an area of influence greater than the single grid box width defined through the gridding process on the 850-hPa surface, we have enlarged the front objects by plus and minus 2 grid boxes. These enlarged areas will be referred to as cold front objects. During testing of the methods, this expansion was found to identify the actual overlap of the front and DI objects (seen through inspection of a number of cases) enabling us to quantify the frequency of the link between the fronts and DIs. This expansion also allows for the fact that most fronts will move during the 6-h time window between data points, so that precipitation can be more fairly attributed to the fronts. The partitioning into different front types is performed *before* the expansion of the front area of influence is applied. This results in the overlapping of central front objects and trailing front objects.

A simple matching procedure is applied to the extended front objects and the DI objects similar to that used in Catto et al. (2015) to match fronts and WCBs. If a particular grid point contains both a front object and a DI object (i.e., there is an overlap of the objects somewhere), then all grid points within those objects are classed as matching.

**Fig. 1:**
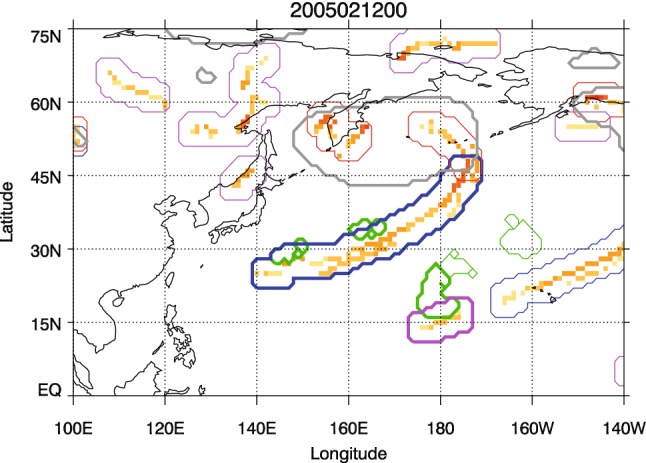
Case study example from 00 UTC, 12 February 2005. Red contours indicate central fronts, blue contours indicate trailing fronts, pink contours indicate isolated fronts, green contours indicate DI objects, and grey contours show the cyclone masks. The gridded cold fronts are shown by the yellow/red grid boxes, with the darker colors indicating stronger wet bulb potential temperature gradients. The thick contours for the front and DI objects indicate that they are matched

To demonstrate the partitioning of the fronts and the matching, the outlines of the three front object types can be seen in Fig. 1, which shows a case from 00 UTC on the 12th February 2005. In the North Pacific there is a deep low pressure system. A cold front associated with this system, shown by the yellow/red grid boxes, can be seen to the east of the low and reaching southwestwards. Front objects matching with DIs (and DI objects matching with fronts) are shown with thick contours and non-matching objects have fine contours. Part of the identified front lies within the closed MSLP contours of the low pressure system, and this part of the front is defined as a central front. The outline of the extended area of this part of the front object can be seen by the red contours surrounding the front. South of this is a section of front that is part of the same front line, but lies outside of the outermost closed MSLP contour, and is therefore defined as a trailing front (enclosed with a blue contour). Further south and quite separate from the low pressure system, a small isolated front can be seen (enclosed with a purple contour). The areas surrounded by the green contours overlapping with the trailing front and the isolated front are DI objects. The trailing front and the isolated front are classed as matching since their object areas overlap with the DI objects.

**Fig. 2:**
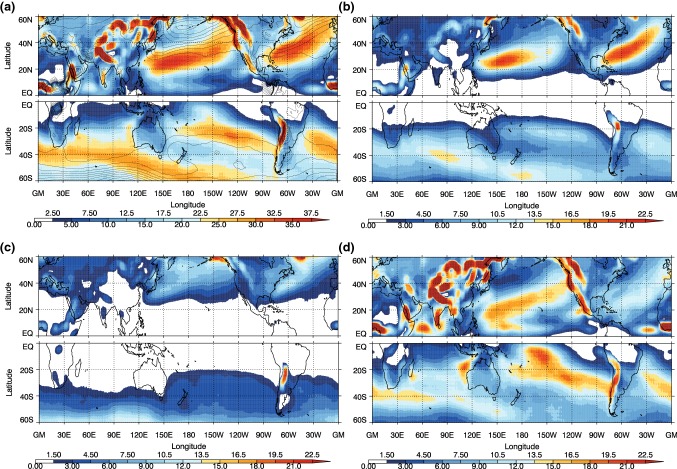
Frequency of cold front objects as a percentage of all 6-hourly analysis times for **a** all fronts, **b** trailing fronts, **c** central fronts, and **d** isolated fronts for NH DJF and SH JJA. Cyclone frequency is shown in **a** as black contours, with contour interval of 5%

**Fig. 3:**
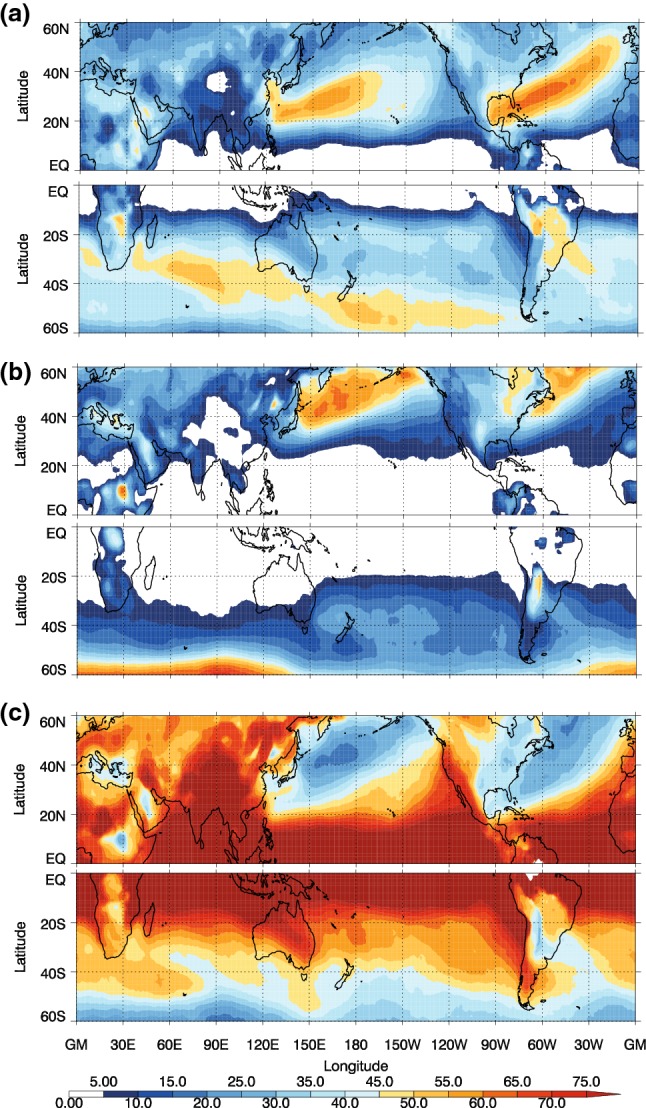
Proportions of different cold front types (%) relative to the total of all fronts for DJF for the NH and JJA for the SH for **a** trailing fronts, **b** central fronts, and **c** isolated fronts

The frequency of the different front types, which is calculated by counting the occurrence of particular front objects at each grid point, for the winter seasons (DJF in the NH and JJA in the SH) can be seen in Fig. 2, while the ratios of each type to all cold fronts are shown in Fig. 3. Figure 2a shows that the highest frequency of fronts occurs equatorward of the main extratropical storm tracks, as previously shown in Berry et al. (2011). There are some features that may not be considered as synoptic fronts, for example the high frequency of fronts over the coast of West Africa is likely associated with a land-sea contrast, and this is also picked up by other front detection methods (Spensberger and Sprenger 2018). Some of the features identified in the tropics may be associated with convergence zones (e.g. Berry and Reeder 2014; Weller et al. 2017). The frequency of front objects shown here in Fig. 2 is generally higher than previous studies using the same front identification method (or other methods), due to the fact that the front lines have been expanded to front objects, and the frequency is the count of the presence of a front object at each grid box. The patterns we find are very similar to the climatologies of Spensberger and Sprenger (2018), but with higher frequencies at lower latitudes than Parfitt et al. (2017a), for example, a common feature of studies including moisture in their thermal variable (Thomas and Schultz 2018a, b).

The trailing fronts occur most frequently on the equatorward side of the main storm tracks (Figs. 2b, 3a), particularly in the NH, consistent with the analysis of trailing cold fronts in Catto and Pfahl (2013). The central fronts generally occur at higher latitudes associated with the path of the MSLP minima identified as cyclones. In the SH winter, there is a maximum in the trailing front frequency spiralling poleward from the South American coast past the south of Africa, and to the south of Australia. There is a second maximum around $20^{\circ }\hbox {S}$ in the central Pacific. The central fronts mostly occur at very high latitudes, associated with the climatological cyclone frequency maximum around the Antarctic coast shown in Fig. 2c and in Wernli and Schwierz (2006). In both hemispheres the isolated fronts occur more frequently in the regions where there are fewest extratropical cyclones identified (e.g., Wernli and Schwierz 2006), giving the highest proportion of total fronts in the low latitudes and on the eastern sides of the oceanic storm tracks (Fig. 3c). Although continental Australia experiences few fronts, relatively these are mostly isolated fronts. The very high frequency of isolated fronts over high topography indicates the influence of the topography on the temperature gradients, and may also be an artefact of including fronts with low frontal speeds. Many isolated fronts are identified in the subtropics, indicating that these fronts are not related to midlatitude systems (Reeder et al. 2000).

## Statistics and characteristics of matched cold fronts and DIs

DIs are most commonly identified during the winter season in each hemisphere, with fewer identified during the other seasons (Raveh-Rubin 2017), so the procedures described above have been applied to the ERA-Interim data for NH DJF and SH JJA. Table 1 shows the total number of fronts and DIs that are identified over the whole period for the whole of the NH during DJF, and the whole of the SH during JJA separately, as well as the percentage of the different types of fronts matched with DIs, and the percentage of DIs matched with the different types of fronts. It is worth noting that the number of “all fronts” is not the sum of the different types of fronts, since at each time point, many fronts will be split into their central and trailing front parts. In total, 62% of winter DIs in the NH and 65% of winter DIs in the SH are matched with fronts, with the highest proportion of these being trailing fronts (33% in the NH and 31% in the SH) and isolated fronts (31% in the NH and 35% in the SH). In the NH 9% of all cold fronts are matched with DIs, with 11% in the SH. This is much higher for trailing fronts at 20% and 19% in the NH and SH respectively.

**Table 1:** Total number of fronts and DIs over the whole of the NH for DJF and the whole of the SH for JJA

Objects	Fronts matched	Fronts not matched	% Matched	DI matched	DI not matched	% Matched
DJF NH						
All fronts	63,326	615,816	9	69,820	42,770	62
Trailing fronts	31,972	124,342	20	36,954	75,636	33
Isolated fronts	31,911	380,398	8	35,147	77,443	31
Central fronts	15,348	201,788	7	16,365	96,225	15
JJA SH						
All fronts	71,286	581,912	11	84,124	45,143	65
Trailing fronts	32,219	140,896	19	40,070	89,197	31
Isolated fronts	38,654	317,407	11	45,758	83,509	35
Central fronts	11,615	230,474	5	13,536	115,731	10

Total number of DIs for DJF is 112,590 and for JJA is 129,267

In the following sections, maps of the matching frequency and the proportion of the total will show the global distribution of the co-occurrence of cold fronts and DIs.

### Global distribution of matched cold fronts and DIs

**Fig. 4:**
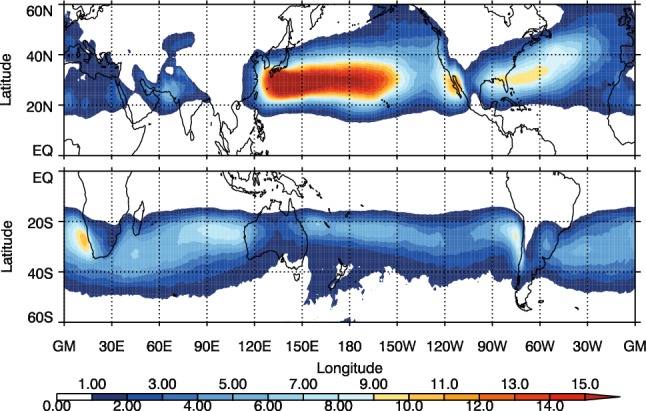
Frequency of occurrence, as a percentage of 6-hourly analysis times, of DI objects for DJF in the NH and JJA in the SH

**Fig. 5:**
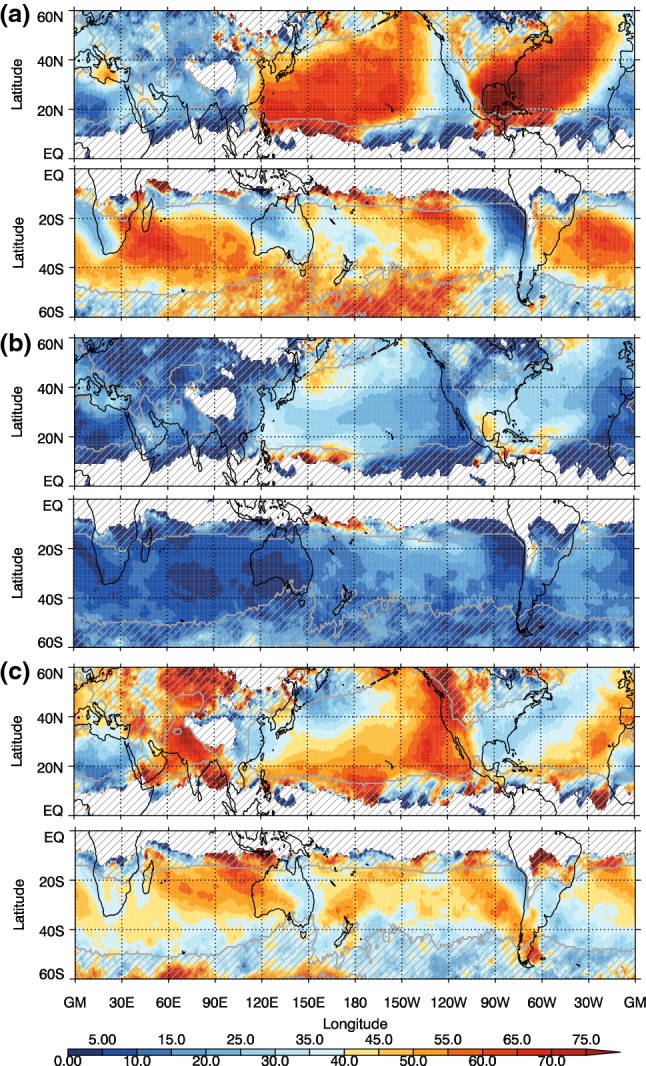
Proportion (%) of DI objects matched with **a** trailing fronts, **b** central fronts, **c** isolated fronts for DJF in the NH and JJA in the SH. Grey hatching shows where the frequency of occurrence of the DIs is less than 1%

A high proportion of DIs match with cold fronts, so here we start by examining the geographical distribution of DI objects (Fig. 4), and how it is modified in the cases of matching with various front types (Fig. 5). The maximum frequency of occurrence of DI objects occurs over the North Pacific storm track of up to 20%, with other lesser maxima over the North Atlantic storm track (around 10%) and over the west coast of the USA. In the SH the highest frequency of DI object occurrence lies in a band between 20° and $40^{\circ }\hbox {S}$, with the highest values to the west of the continents of around 11%. The patterns of DI frequency look very similar to the counts of DI trajectories 24 to 48 h after descent begins (shown in Figures 3 and 4 in Raveh-Rubin 2017). This is consistent with the requirement that the DI trajectory must be below 700 hPa (and therefore must have already descended quite far) to be part of a DI object in the present study. In the present study, the occurrence frequencies are slightly higher than those in Raveh-Rubin (2017). This stems from the process employed to generate two-dimensional DI objects, which takes into account DIs at multiple relative times from the start of descent, as long as they lie below the 700-hPa height. In Raveh-Rubin (2017), the frequency of occurrence statistics consider only single relative time steps, resulting in lower trajectory counts.

Since the three front types occur in rather distinct geographical regions, the DIs that match with each type of front are also preferentially located in different regions (Fig. 5). DIs matching with trailing fronts are located equatorward of the main storm tracks, but covering an area extending beyond the highest trailing front frequencies (compare Fig. 5a with 3a). A strong east–west dipole emerges in the main ocean basins in both hemispheres. While over 70% of DIs occurring in the central and western ocean basins match with trailing fronts, less than 30% do so in the eastern parts. A mutually exclusive pattern exists for DIs matching with isolated fronts, such that a low proportion of matching exists in the western ocean basins, and a high proportion in the east (Fig. 5c). An exception to this pattern is the area of increased DI frequencies west of the Andes, which do not match with any type of front. Matching of DIs with central fronts occurs in localized coastal regions (Fig. 5b), which indeed sums up to the lowest matching proportion of the front types (Table 1).

**Fig. 6:**
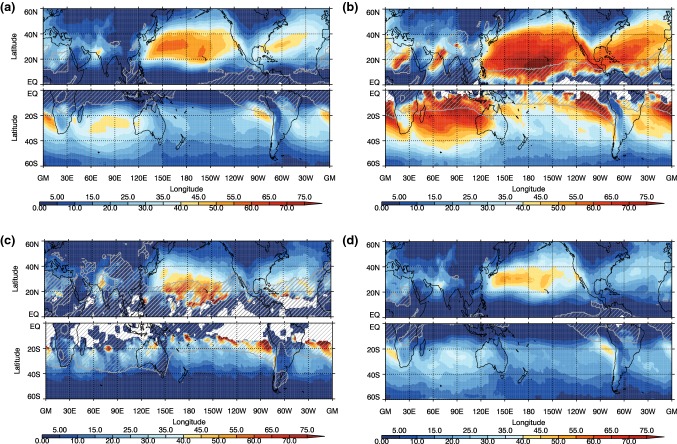
Proportions of different cold front types that are matched to DIs out of all fronts of the same type for DJF in the NH and JJA in the SH for **a** all fronts, **b** trailing fronts, **c** central fronts, and **d** isolated fronts. Grey hatching shows where the frequency of the front type is below 5% for all fronts or 2% for the individual front types

We consider now the proportion of each of the front types that are matched to DIs (Fig. 6). Figure 6a indicates that the highest proportion of matching for all cold fronts occurs south of the major NH storm track regions (over the North Atlantic and North Pacific Oceans), and in a latitude band between $20^{\circ }$ and $40^{\circ }\hbox {S}$, with maxima in the eastern ocean basins. In the NH the maximum reaches around 60% of cold fronts matching with DIs in the North Pacific region. This is consistent with the highest frequency of DIs occurring in this region (Fig. 4). Overall the highest proportion of matching for all fronts corresponds closely to the pattern of DI object frequency, suggesting that the matching of fronts is limited by the DI occurrence (see also Table 1).

Very high proportions of trailing fronts are matched with DIs (Fig. 6b) in both the NH and SH with maxima above 75%. The highest proportions occur at lower latitudes than for all fronts. In the NH there is also a distinctive southwest to northeast tilt to the pattern in both the NH storm track regions. These features indicate that many cyclones propagating along the major storm track axes that have trailing cold fronts will be associated with a DI. This is also consistent with DIs being potentially important for the equatorward propagation and reach of these trailing fronts. In the SH, the highest proportions of trailing fronts that match with DIs are seen in the Indian Ocean sector, north of $20^{\circ }\hbox {S}$, to the west of South America, and to the west of South Africa. Interestingly, trailing front frequencies are lowest in the eastern ocean basins in the SH, but when they do occur there, it is often in association with a DI (Figs. 2b, 3a, 6b). Yet, isolated fronts are a more common type of front in the eastern ocean basins, thus making up the largest proportion there (Fig. 5c).

The pattern for the proportion of central fronts matched to DIs (Fig. 6c) indicates a lower overall matching proportion. Although the frequency of central fronts in the SH regions where DIs occur during JJA is very low (Fig. 2) and the proportion of DIs matched with central fronts is low, up to 35% of central fronts are matched with DIs in the SH where the central front frequency is above 2%.

The isolated fronts matched with DIs (Fig. 6d) follow a similar pattern to that of all fronts with maxima across the regions of highest DI frequency. Despite due to the low frequency of occurrence of DIs at low latitudes, there are low proportions of isolated fronts matched with DIs at these low latitudes (Fig. 6d). Maxima occur off the west coast of South America and South Africa, over the north Pacific ocean (between 20° and 40°N), and over the Indian Ocean. Supplementary Figure S4 shows that the frequency of isolated fronts is quite sensitive to the minimum length requirement of 5 grid boxes. However, the frequency of isolated fronts matching with DIs is not. The proportion of trailing and isolated fronts associated with DIs is slightly lower when the minimum length criterion is not applied (Supplementary figure S8), but is insensitive to the search radius parameter used in the front identification.

### Statistical distributions of front intensities

In order to determine the importance of the link between cold fronts and DIs on the characteristics of fronts, we have determined the statistics of the strength of matched and non-matched fronts (defined as the gradient of $\theta _{w}$ across the front). The strength is known at each point along a front, and for each front object the maximum strength within the object is used to determine the strength of that front. For all the following statistics, only fronts that have their maximum strength between 0° and $60^{\circ }\hbox {N}$ and between 0° and $60^{\circ }\hbox {S}$ are considered. This excludes the many fronts that are found around the Antarctic coast, which are associated with the strong temperature gradients at the edge of the continent, and have rather different environments. We have tested the sensitivity to choosing the average strength over the front instead of the maximum strength, and find the conclusions unchanged.

Table 2 shows the average maximum front strength for the different types of fronts in the NH and Table 3 the SH. Considering all fronts together (before the fronts are separated into central, trailing, and isolated fronts), matched fronts tend to be 26% stronger in the NH and 23% stronger in the SH than non-matched fronts. All types of fronts are stronger in the NH than in the SH, consistent with Naud et al. (2015). Matched central fronts are the strongest types of fronts in both hemispheres, which could be expected due to their location closest to the strong baroclinic zone indicated by the maxima in cyclone frequency (Fig. 2). Matched central fronts are 22% stronger in the NH and 11% stronger in the SH compared to non-matched central fronts, but the matching of this type occurs most rarely (Table 1). The matching of trailing fronts with DIs gives a smaller difference, with matched trailing fronts 13% stronger in the NH and 17% stronger in the SH on average, however, trailing fronts match most commonly with DIs, out of all front types. Finally, isolated fronts in the NH are 16% stronger when matched with DIs, and 19% stronger in the SH.

**Table 2:** Front strength (gradient of 850-hPa wet bulb potential temperature across the front) for matched and non-matched fronts for NH DJF

Front type	Gradient matched	Gradient not matched	% Difference
All fronts	2.12	1.68	26
Trailing fronts	2.16	1.91	13
Isolated fronts	1.79	1.54	16
Central fronts	2.43	1.99	22

Only fronts with their maximum gradient between 0° and $60^{\circ }\hbox {N}$ are included in the statistics. Gradient is the mean over all fronts of the maximum gradient along each front (*K* / 100 km). All differences are statistically significant at the 95% level

**Table 3:** Front strength (gradient of 850-hPa wet bulb potential temperature across the front) for matched and non-matched fronts for SH JJA

Front type	Gradient matched	Gradient not matched	% Difference
All fronts	1.81	1.47	23
Trailing fronts	1.90	1.63	17
Isolated fronts	1.59	1.34	19
Central fronts	1.91	1.72	11

Only fronts with their maximum gradient between 0° and $60^{\circ }\hbox {S}$ are included in the statistics. Gradient is the mean over all fronts of the maximum gradient along each front (*K* / 100 km). All differences are statistically significant at the 95% level

**Fig. 7:**
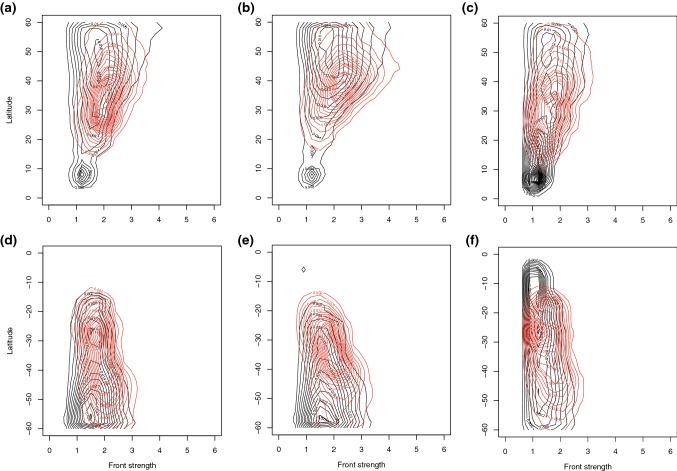
Two-dimensional probability density of the maximum gradient of $\theta _w$ within non-matched (black contours) and matched (red contours) **a**, **d** trailing fronts, **b**, **e** central fronts, **c**, **f** isolated fronts binned according to the latitude at which the maximum gradient occurs, for DJF in the NH (**a**–**c**) and JJA in the SH (**d**–**f**). Units of front strength are *K* / 100 km. Latitude bins are $2^{\circ }$ and gradient bins are $0.6\,K/100$ km, while contour intervals are 0.002

We have already shown above that the different front types occur at different latitudes, and that the maximum frequency of matching with DIs is not necessarily at the same latitude as the maximum front frequency. In addition, previous work has shown that front strength varies with latitude (Catto et al. 2014). In order to investigate whether the statistics indicated above are due to differences in the characteristics of the matched and non-matched fronts, or simply an artefact of the latitude at which matching occurs, Fig. 7 shows the two-dimensional density function of front strength with latitude, plotted using a kernel density function with the bins detailed in the figure caption.

For all front types and in both hemispheres, the maximum front strength for both matched and non-matched fronts increases with latitude (Fig. 7, consistent with Catto et al. (2014)). The fronts tend to be stronger in the NH than the SH, consistent with the findings of Naud et al. (2015). Another feature that is common to the different front types in the NH is the maximum density in non-matched fronts at around $5^{\circ }\hbox {N}$. Inspection of the maps of cyclone frequency suggest this is associated with the fronts identified near the coast of West Africa. DI trajectories do not tend to reach this latitude by 48 h from descent (Raveh-Rubin 2017) so none of these fronts appears to be matched with DIs.

Matched trailing fronts in the NH (red contours in Fig. 7a) are most common between 20° and $40^{\circ }\hbox {N}$, and at those latitudes the distribution of front strength lies shifted to stronger fronts compared to the non-matched fronts. The same is true in the SH (Fig. 7d), with the front strengths stronger at all latitudes for the matched fronts compared to the non-matched fronts. Central fronts (Fig. 7b, e) show similar characteristics to trailing fronts. In the NH there is a peak in density for the matched central front strength at $40^{\circ }\hbox {N}$ with strength of greater than $2\,K/100$ km. There is also a high density in the matched central fronts around $30^{\circ }\hbox {S}$ with strength of $2\,K/100$ km. Isolated fronts (Fig. 7c, f) tend to occur at lower latitudes, with the matched cases peaking around $30^{\circ }\hbox {N}$ and S. Again, in both hemispheres the matched fronts have higher peak front strengths at all latitudes.

The rest of the front characteristics will be considered for only the trailing and isolated fronts since these appear to be quite different, and occupying different regions of the globe.

### Statistical distributions of front size

**Fig. 8:**
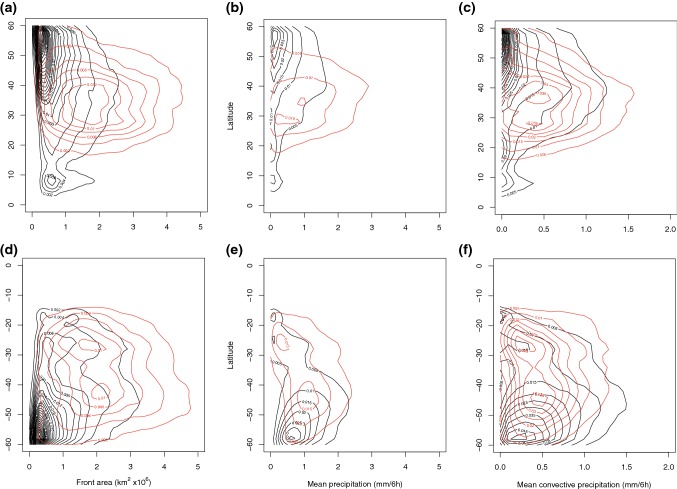
Two-dimensional probability density of properties of non-matched (black contours) and matched (red contours) *trailing* fronts for **a**, **d** front area, **b**, **e** mean precipitation, **c**, **f** mean convective precipitation binned according to the latitude at which the maximum gradient occurs, for DJF in the NH (**a**–**c**) and JJA in the SH (**d**–**f**). Precipitation is calculated as the mean over the front object area. Latitude bins are $2^{\circ }$, precipitation bins are 0.1 mm/6 h with contour intervals of 0.005 and area bins are $0.1\,\text {km}^{2}\times 10^{6}$ with contour intervals of 0.002

On visual inspection of case studies of matching and non-matching fronts, a feature that seems to differ is the length (or area) of the fronts with or without matched DIs. This has also been investigated statistically with the mean front area of the expanded front object shown in Table 4, and the two-dimensional histograms of front area against latitude shown in Figs. 8a, d (trailing fronts) and 9a, d (isolated fronts). For both hemispheres and for both trailing and isolated fronts, there is a clear large mean positive difference in front area when associated with DIs, in some cases more than double for matched fronts compared to non-matched fronts (Table 4). For trailing fronts the highest density for matched fronts occurs around $2\times 10^{6}\,\hbox {km}^2$ at $40^{\circ }\hbox {N}$ and at $40^{\circ }\hbox {S}$ and $25^{\circ }\hbox {S}$. For the isolated fronts matched with DIs the peak density is at much smaller front areas (close to $0.5\times 10^{6}\,\hbox {km}^2$) at $30^{\circ }\hbox {N}$ and S, but there is a much broader spread of front areas for the matched fronts, going up to $4\times 10^{6}\,\hbox {km}^2$.

**Table 4:** Properties of matched and non-matched fronts for NH (0–$60^{\circ }\hbox {N}$) DJF and SH (0–$60^{\circ }\hbox {S}$) JJA for trailing fronts and isolated fronts with matched DIs and without matched DIs

Season and front	*A* DI	*A* noDI	*P* DI	*P* noDI	*CP* DI	*CP* noDI
DJF trailing	2.06	0.94	1.47	0.96	0.56	0.40
JJA trailing	2.33	1.17	1.42	1.32	0.56	0.61
DJF isolated	1.35	0.77	0.76	0.60	0.30	0.27
JJA isolated	1.62	0.92	0.92	0.88	0.37	0.47

Front object area (*A*; km$^{2} \times 10^{6}$), mean precipitation over the front object area (*P*; mm/6 h), and mean convective precipitation over the front object area (*CP*; mm/6 h)

**Fig. 9:**
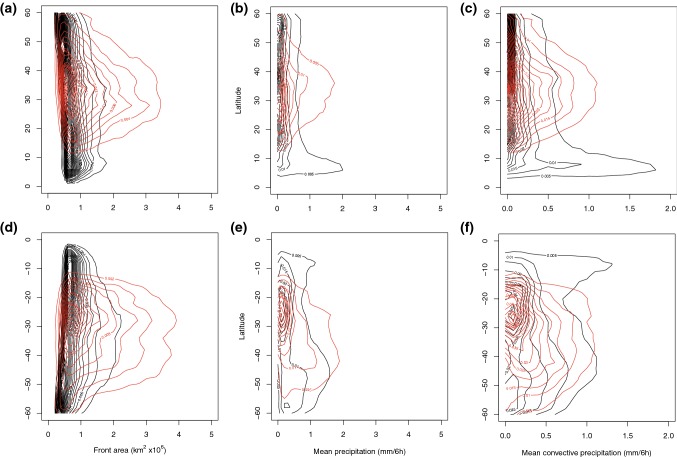
Two-dimensional probability density of properties of non-matched (black contours) and matched (red contours) *isolated* fronts for **a**, **d** area, **b**, **e** mean precipitation, **c**, **f** mean convective precipitation binned according to the latitude at which the maximum gradient occurs, for DJF in the NH (**a**–**c**) and JJA in the SH (**d**–**f**). Precipitation is calculated as the mean over the front object area. Latitude bins are $2^{\circ }$, precipitation bins are 0.1 mm/6 h with contour intervals of 0.005, and area bins are $0.1\,\text {km}^{2}\times 10^{6}$ with contour intervals of 0.002

### Statistical distributions of frontal precipitation

**Fig. 10:**
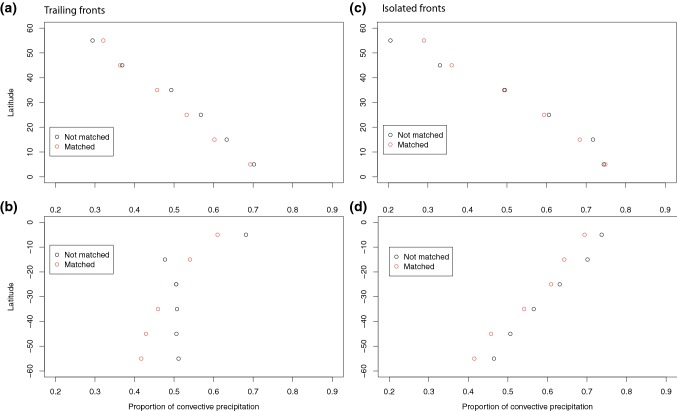
Variation with $10^{\circ }$ latitude band of the proportion of convective precipitation for fronts matched with DIs (red) and not matched with DIs (black) for **a** trailing fronts in the NH DJF, **b** trailing fronts in the SH JJA, **c** isolated fronts in the NH DJF, and **d** isolated fronts in the SH JJA

Clearly at all latitudes there is an increase in front strength when fronts are matched with DIs (Sect. 3.2). In previous work extreme precipitation events were found to be associated with fronts that are up to 30% stronger than for less extreme precipitation events (Catto and Pfahl 2013). The influence of DIs on front strength may, therefore, also contribute to increasing the precipitation associated with the fronts. We have calculated the average precipitation within the front object area referred to above. This area (using the expanded front objects) is comparable to the search area used to define frontal precipitation in Catto et al. (2012); Catto and Pfahl (2013). Table 4 shows the mean precipitation and mean convective precipitation associated with matched and non-matched trailing and isolated fronts. In the NH during DJF, where the largest influence of the matching of DIs can be seen, the mean precipitation associated with trailing fronts matched with DIs is 1.5 times that without a DI (1.47 mm/6 h and 0.96 mm/6 h respectively), and the convective precipitation is 1.4 times as large (0.56 and 0.40 mm/6 h). This decreases slightly the proportion of total precipitation that comes from convection in the case of matched trailing fronts in the NH from 42% to 38% (since the total precipitation is greater). When considering the average proportion of convective precipitation in latitude bands of $10^{\circ }$, Fig. 10a reveals that the proportion of convective precipitation is greater for non-matched trailing fronts in most latitude bands. Figure 8b, c, e, f shows the two dimensional histograms of total precipitation and convective precipitation for trailing fronts. In the NH the larger total precipitation associated with matched fronts can be clearly seen, with peaks in the density around $35^{\circ }\hbox {N}$ and at 1 mm/6 h, while the peak density for non-matched trailing fronts occurs near $60^{\circ }\hbox {N}$ with very low precipitation values. There are higher precipitation values for matched fronts at all latitudes in the NH, seen by the extension of the red curves to much higher precipitation intensities.

In the SH there are smaller differences in the mean precipitation values between matched and non-matched trailing fronts (1.42 mm/6 h and 1.32 mm/6 h respectively), and the convective precipitation is even slightly greater for the non-matched fronts. However, the two-dimensional histograms show a more complex picture, with the matched trailing fronts having higher total and convective precipitation between 20° and $40^{\circ }\hbox {S}$, but lower values between 45° and $60^{\circ }\hbox {S}$. This may explain the smaller SH average precipitation difference compared to the NH. The highest density for the non-matched trailing front mean precipitation can be seen close to $60^{\circ }\hbox {S}$, with a value of around 0.6 mm/6 h, whereas the peak for the matched trailing fronts is at $45^{\circ }\hbox {S}$ at over 1 mm/6 h. There is also a second peak density at $25^{\circ }\hbox {S}$ with lower precipitation values. This double peak structure can also be seen for the convective precipitation (Fig. 8f) but with intensities around half of the total precipitation.

Isolated fronts in the NH during DJF show, on average, a much weaker influence of the matching with DIs but total and convective precipitation are both larger for the matched fronts. Figure 9b shows that the peak density for precipitation in non-matched isolated fronts is around zero over a broad latitude band, and in fact the median value is 0.23 mm/6 h over all latitudes. There is a maximum in precipitation shown equatorwards of $10^{\circ }\hbox {N}$, which is not seen for the matched isolated fronts since the frequency of matching at this latitude is very small. Over the latitude band of 20°–$50^{\circ }\hbox {N}$, the total and convective precipitation (Fig. 9b, c) are higher for the matched isolated fronts, but the peak density is still close to zero (a median of 0.31 mm/6 h for mean precipitation).

In the SH, a similar picture for isolated as trailing fronts can be seen, with higher mean total precipitation for matched fronts compared to non-matched fronts, but lower mean convective precipitation (Table 4). The two dimensional histograms (Fig. 9e, f) show some similarities with the trailing fronts in the SH, but are quite different to the NH. The peak density in total and convective precipitation can be seen around $25^{\circ }\hbox {S}$ and is very similar for the matched and non-matched fronts. Since precipitation is averaged within the front area, the combined increase of front area and mean precipitation for matched fronts, indicates that total precipitation amounts are even larger, compared to non-matched cases.

The variation with latitude of the proportion of convective precipitation (Fig. 10) reveals that it generally increases towards lower latitudes for trailing and isolated fronts as we would expect from the global distribution of convection. For both types of fronts and in almost all latitude bands, the fronts not matched with DIs have higher convective precipitation proportions. So, while the association with DIs shows climatologically higher total and convective precipitation over the fronts, the increase in total precipitation is greater than the increase in convective precipitation when DIs are present.

### Characteristics of fronts in different DI regions

**Fig. 11:**
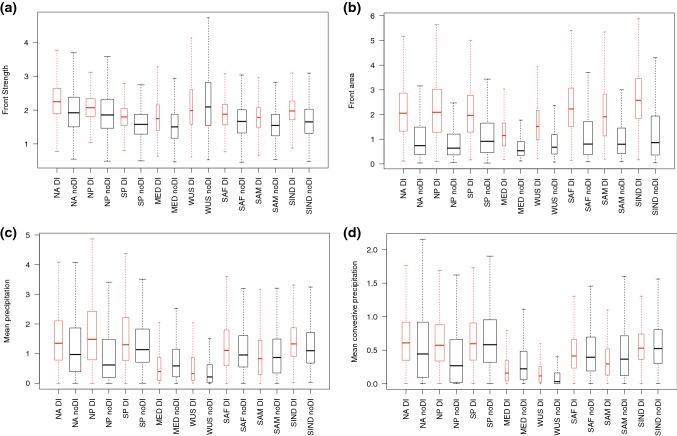
Box plots showing *trailing* front characteristics with (in red) and without (in black) matched DIs for the regions defined in Raveh-Rubin (2017). Whiskers show $1.5 \times \text {IQR}$. The width of the box is proportional to the square root of the number of fronts in each region. Regions are defined as follows: North Atlantic (NA), $20^{\circ }$–$60^{\circ }\hbox {N}$, $90^{\circ }$–$20^{\circ }\hbox {W}$; North Pacific (NP), $20^{\circ }$–$60^{\circ }\hbox {N}$, $100^{\circ }\hbox {E}$–$150^{\circ }\hbox {W}$; South Pacific (SP), $60^{\circ }$–$10^{\circ }\hbox {S}$, $160^{\circ }\hbox {E}$–$110^{\circ }\hbox {W}$; Mediterranean (MED), $20^{\circ }$–$60^{\circ }\hbox {N}$, $50^{\circ }\hbox {W}$–$35^{\circ }\hbox {E}$; Western United States coast (WUS), $20^{\circ }$–$60^{\circ }\hbox {N}$, $125^{\circ }$–$110^{\circ }\hbox {W}$; Southwestern Africa (SAF), $60^{\circ }$–$20^{\circ }\hbox {S}$, $30^{\circ }\hbox {W}$–$20^{\circ }\hbox {E}$; Western South America (SAM), $60^{\circ }$–$20^{\circ }\hbox {S}$, $100^{\circ }$–$60^{\circ }\hbox {W}$; South Indian Ocean (SIND), $60^{\circ }\hbox {S}$–$0^{\circ }$, $50^{\circ }$–$100^{\circ }\hbox {E}$

**Fig. 12:**
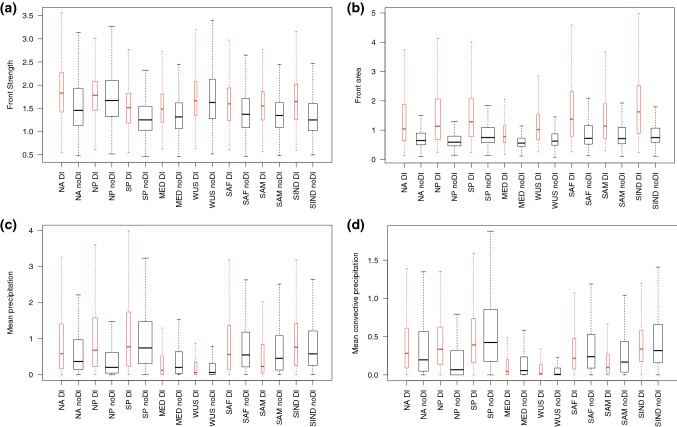
Box plots showing *isolated* front characteristics with (red) and without (black) matched DIs for the regions defined in Raveh-Rubin (2017). Whiskers show $1.5 \times \text {IQR}$. The width of the box is proportional to the square root of the number of fronts in each region. See Fig. 11 for region definitions

Raveh-Rubin (2017) identified distinct characteristics of the DIs in different regions, which were ultimately reduced to “storm-track” or “non storm-track” DIs. Here we have investigated the regional differences in the front characteristics in these same regions (Figs. 11, 12), to examine whether matches of DIs and fronts in the storm track regions have different impacts to similar matches outside of the main storm tracks. Here we highlight the main results of this investigation.

For trailing fronts, the differences between fronts matched and not matched with a DI are consistent across the storm track regions (NA, NP, SP), with higher front strength, area, precipitation, and convective precipitation for matched fronts, (shown by boxplots in Fig. 11). In fact, almost all regions show stronger fronts with DIs, except the WUS region. All regions show larger front area, with much higher variability (shown by the whiskers) for fronts matched with DIs. The Mediterranean is the only region in which the mean precipitation for fronts with DIs is lower than for fronts without DIs, which is consistent with the very dry DI trajectories in this region (Raveh-Rubin 2017). WUS trailing fronts have some of the highest front strengths, but the lowest associated precipitation, possibly related to these fronts being at the end of the storm track and no longer experiencing strong frontogenesis.

The isolated fronts have the median values of front strength and area higher for fronts matched with DIs in all regions (Fig. 12). Considering the mean precipitation, the SH regions tend to show either smaller differences between matched and non-matched isolated fronts, or higher precipitation for the non-matched fronts. This is consistent with the 2D histograms shown in Fig. 9 and indicates that it is common across the hemisphere.

**Fig. 13:**
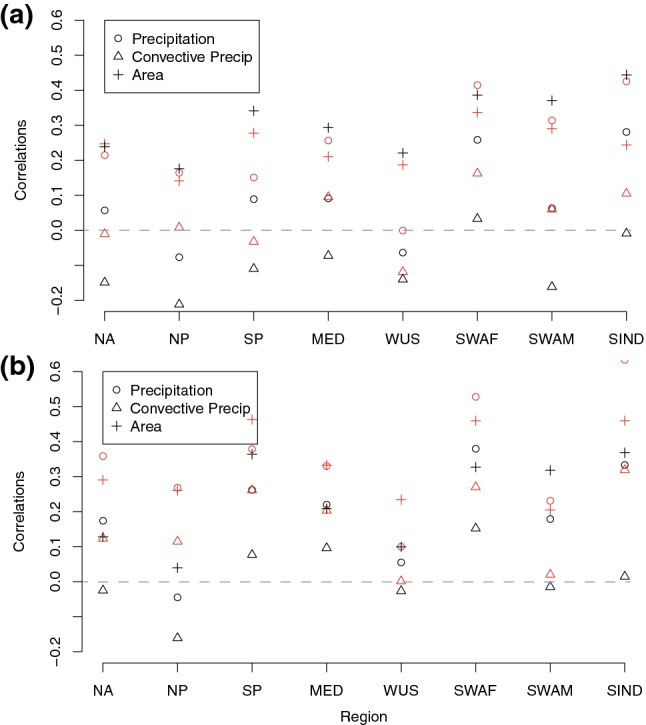
Correlation between **a** trailing and **b** isolated front strength and the other front characteristics for the regions defined in Raveh-Rubin (2017) for matching fronts (red) and non-matching fronts (black). Dashed horizontal line shows the zero correlation

The boxplots indicate that the characteristics of the fronts do not necessarily all vary together, so to investigate the co-variability and how this is influenced by the association with a DI, Fig. 13 shows the correlations between front strength and the three other front characteristics (precipitation, convective precipitation, and area). Correlations between the strength of trailing front without DIs and their associated mean precipitation are positive everywhere except the NP and WUS regions. For the positive correlations, they are further enhanced in all regions by the matching with DIs. Convective precipitation is mainly negatively correlated with front strength for the non-matched fronts, and weakly positively correlated for the matched fronts. The correlations between front strength and area are also positive everywhere for the trailing fronts, but for most regions, this correlation is higher for the non-matched fronts. This indicates that without the presence of a DI, when mean frontal area is comparatively smaller, there is a stronger tendency for strong fronts to be larger.

The isolated fronts show mostly the same features as the trailing fronts, i.e., more positive correlations between front strength and precipitation for matched fronts. However, the opposite effect to that for trailing fronts is seen for the front area, with higher correlations between front strength and area for the matched fronts in all regions except SWAM, indicating that the presence of a DI contributes both to stronger and larger fronts.

## Discussion and conclusions

### Summary

The global and long-term (1979–2014) frequency and distribution of the co-occurrence of cold fronts and DIs have been investigated for the first time by using an automated front climatology and a Lagrangian definition of DIs. The cold fronts were separated into three types; central fronts that occur within a cyclone area, trailing fronts that are outside of any cyclone area but are connected to central fronts, and isolated fronts that are not linked to cyclones. This separation has revealed distinct spatial distributions and characteristics and has allowed the analysis of the link between the cold fronts and DIs in the context of different front types. The global spatial distribution of the matching of cold fronts and DIs has been investigated, as well as a statistical analysis of four characteristics of the fronts: front strength, front area, average front precipitation, and average front convective precipitation. A summary of the main findings of the study that answer the questions posed in the introduction, and accompanying discussion of several aspects of the study are given below.

1. Central fronts occur most frequently in the major storm track regions of both hemispheres. Trailing fronts are most frequently located on the equatorward edge of the storm tracks in the NH with a southwest to northeast tilt, and in a spiral band from South America across the Atlantic and Indian Oceans to the south of Australia and New Zealand, as well as in the South Pacific Convergence zone region in the SH. Isolated fronts are more prevalent at lower latitudes and towards the eastern ends of the storm tracks, with most of the cold fronts occurring between $20^{\circ }\hbox {N}$ and $20^{\circ }\hbox {S}$ being isolated fronts.
2. Trailing fronts are most often matched with DIs in both hemispheres compared to the other two front types, with 20% being matched in the NH, and 19% in the SH. Isolated fronts are matched to DIs 8% and 11% of the time in the NH and SH respectively, and central fronts only 7% and 5%. This corresponds to roughly a third of DIs matching with trailing fronts, and another third matching with isolated fronts. Thus, half of the DIs that match with cold fronts indeed fit the conceptual picture of a DI conveyor belt to the rear of a cyclone and its trailing cold front (Browning 1997). Yet, the other half of matched DIs are associated with fronts that are not related to a cyclone.
3. In low latitudes, where the DIs are infrequent (occurring less than 1% of the time), a very high proportion of trailing fronts are associated with DIs. This shows that in these regions DIs and trailing fronts almost always occur together, suggesting that the presence of a DI could be essential for producing a trailing front in the region. Testing this hypothesised causality will be the subject of a future study.
4. For all front types, the mean maximum front strength (defined as the maximum gradient of wet bulb potential temperature across the front within the front area) is higher for fronts matched with a DI (Tables 2, 3). This can be seen across all latitudes, and in the regions defined in Raveh-Rubin (2017) (Figs. 7, 11, 12). Given the very low moisture content of DI trajectories (Raveh-Rubin 2017), and the sensitivity of wet bulb potential temperature to moisture gradients, this is what we would expect.
5. Trailing fronts and isolated fronts that are matched with DIs are larger (or longer) than those not matched, with the average trailing front area double (NH) or almost double (SH) the size when matched, and isolated fronts around 75% larger (Table 4). This is consistent with the idea that the DIs act to lengthen these types of fronts by spreading out behind the cold fronts, providing both a dynamical mechanism to stretch the front, and also delivering cooler, drier air into the region.
6. On average, the mean precipitation across the front area is larger for fronts that are matched with DIs for trailing and isolated fronts, in both the NH and the SH. This difference is clearest in the 2D histograms for the NH (Figs. 8, 9), but also varies across the storm track and non storm track regions. The correlation between front strength and mean precipitation is generally stronger when a DI is present. The average convective precipitation is larger for fronts matched with DIs in the NH (for all regions except the MED), but larger for non-matched fronts in the SH (consistent across the SH regions) (Fig. 13).

### Discussion

Here we have been able to show that all types of fronts that are associated with DIs are stronger in terms of their wet bulb potential temperature gradient than other fronts. This makes sense when we consider the conceptual picture of DIs descending behind the cold fronts and bringing air with very low $\theta _{w}$ (Browning 1997; Raveh-Rubin 2017). However, we have not been able to explicitly show a causal relationship. It may be that the stronger fronts are also associated with stronger cyclones, and therefore are more likely to be associated with DIs. The compositing methodology used in Part 2 (Raveh-Rubin and Catto 2019) will go some way to addressing these interrelationships between strength and DIs.

We have only found two other studies that investigated the size of observed cold fronts. Utsumi et al. (2014) found that cold fronts around Japan are, on average, longer during winter than during summer. Simmonds et al. (2011) also found that fronts are longer during winter in most regions of the SH, and the longest fronts occur in the South Indian Ocean region. Our results clearly show that fronts associated with DIs are larger (i.e., longer) than those not associated with DIs, with this difference being particularly marked in the South Indian Ocean region (Fig. 12). Our hypothesis is that the deformation flow produced by the descending DI trajectories acts to elongate the fronts. This will be further investigated using more targeted sampling of the fronts in future studies.

Schultz (2018) points out that there are a number of different types of cold fronts, such as split fronts, and rearward sloping fronts, and patterns of associated rainfall. In this study we have not attempted to distinguish these, but consider this an interesting avenue for future research using these datasets. Here we have performed some subsetting of the fronts into trailing, central and isolated fronts so that sensible distinctions may be made in the statistics and (in part 2) the spatial patterns. However we have maintained large enough samples of different fronts, which are required in order to be able to generalize and produce robust statistics (Naud et al. 2018). The separation into the different types reveals interesting aspects of the front climatology, such as the high frequency of isolated fronts over the subtropics in both the NH and the SH, and the high proportion of isolated fronts over the subtropical interior of Australia (e.g., the “dryline”, Arnup and Reeder 2006). The use of the wet bulb potential temperature as the thermal variable for the identification of fronts results in more features in lower latitudes than a dry thermal variable may (Thomas and Schultz 2018a, b). As suggested by Catto et al. (2012), the fronts identified by the automated methods in these low latitude regions are not necessarily fronts associated with synoptic scale cyclones, but they certainly are associated with the DIs that form part of the conceptual model of midlatitude cyclones, indicating the importance of the link with DIs in both the mid- and lower-latitudes.

There are some interesting differences in the distributions of precipitation for the trailing and isolated fronts. These may be associated with the different mechanisms influencing precipitation along the fronts. For example, the isolated fronts tend to have higher proportions of convective precipitation than the trailing fronts, consistent with their spatial separation from the major baroclinic zones. The DIs may be triggering or suppressing precipitation depending on whether it is undercutting or overriding the low-level front.

### Further remarks

In this study we have chosen to use the ERA-Interim precipitation due to its global coverage, and long time period. There are, of course, uncertainties associated with using this product due to the precipitation being a modeled field. Recently Naud (2018) compared extratropical cyclone-related precipitation from a number of different precipitation products, including satellite and gauge-based datasets. They found that ERA-Interim estimates lie within the uncertainties of the observational datasets. We can also make an approximate comparison with the previous studies of Catto et al. (2012) and Catto et al. (2013), where GPCP 1 degree daily precipitation was used to estimate the intensity of frontal precipitation. They found the annual average intensity of frontal precipitation in the midlatitudes to be around 5.3 mm/day, and for cold fronts specifically 3.3 mm/day. Despite the differences in the methodology used to calculate these values, they are somewhat comparable to the average front area precipitation found in this study, giving us confidence that the ERA-Interim precipitation can be adequately used to compare the DI and non-DI cold front precipitation.

Here we have used the ERA-Interim reanalysis dataset for the period 1979–2014. There is evidence that the change in sea surface temperature (SST) resolution over the period of the ERA-Interim reanalysis (from $1^{\circ }$ to $0.5^{\circ }$ in 2001, and to $0.05^{\circ }$ in January 2009) has an impact on the frequency of fronts and the mean atmospheric state in the Gulf Stream and Kuroshio Current region (Parfitt et al. 2017b; Masunaga et al. 2015). By comparing the 5-year high-resolution period of 2009–2014 to a number of randomly selected 5-year low-resolution periods (Parfitt et al. 2017b), and using our own front identification, we find (consistent with these studies) that during the high resolution period there is a higher front frequency and DI frequency near to the strongest SST gradients in the NH, and lower front frequency over the low latitudes and over land (not shown). To investigate whether this difference has an impact on our conclusions, we calculated many of the results using the shorter period of 1979–2008. The maps showing the distributions of front and DI frequency and proportion of matched objects are indistinguishable, indicating that our conclusions are insensitive to the SST resolution change. Supplementary Figure S1 shows the characteristics of the matched and non-matched fronts for the two different periods, which indicate no change to the conclusions of the paper.

The climatology presented in this paper, which aims to serve as a guide for physical understanding of the identified features, is based on a number of choices and assumptions in the methods. The sensitivity to some of these choices is quantified in the supplementary material. For example, changing the parameters of the line-joining algorithm (Figures S2–S8), or the *TFP* threshold (Figures S9–S10) results in different front frequencies, and while this changes the proportion of fronts associated with DIs (Figure S11), it does not change the difference between front characteristics with and without DIs. Moreover, trailing fronts are still found to be the most strongly associated with DIs. Identifying fronts on different levels shifts the maximum front frequencies to different locations (Figures S9–S10), and at 925 hPa gives stronger fronts without DIs than with DIs (Figure S12), likely due to the strong temperature contrasts over orography. There are some differences to the isolated front precipitation when identifying the fronts on 700 hPa, which may be associated with differences in the vertical structure of these features (see Raveh-Rubin and Catto 2019). While the exact values change for different parameter choices, overall the conclusions of the study are strengthened by this sensitivity analysis.

In this part of this two-part study, we have examined the statistical properties of the three types of cold fronts, with a particular focus on trailing and isolated fronts. Part II of this study (Raveh-Rubin and Catto 2019) will address the dynamical aspects of the trailing and isolated fronts using composites that show the spatial pattern of variables of interest associated with the environment of the fronts and their impacts.

## Electronic supplementary material

Below is the link to the electronic supplementary material.

Supplementary material 1 (PDF 25656 kb)
