# Supplementary material for: Climatology and dynamics of the link between dry intrusions and cold fronts during winter. Part I: global climatology
Source: Clim Dyn. 2019 May 7;53(3):1873–92. doi: 10.1007/s00382-019-04745-w (PMC6647398; doi:10.1007/s00382-019-04745-w)
Supplement: Supplementary file 1 — Supplementary material 1 (PDF 25656 kb) [file 382_2019_4745_MOESM1_ESM.pdf]

Climatology and Dynamics of the Link Between Dry Intrusions and Cold Fronts During Winter. Part I: Global Climatology

Supplementary Material

Jennifer L Catto and Shira Raveh-Rubin

1 Supplementary Figures

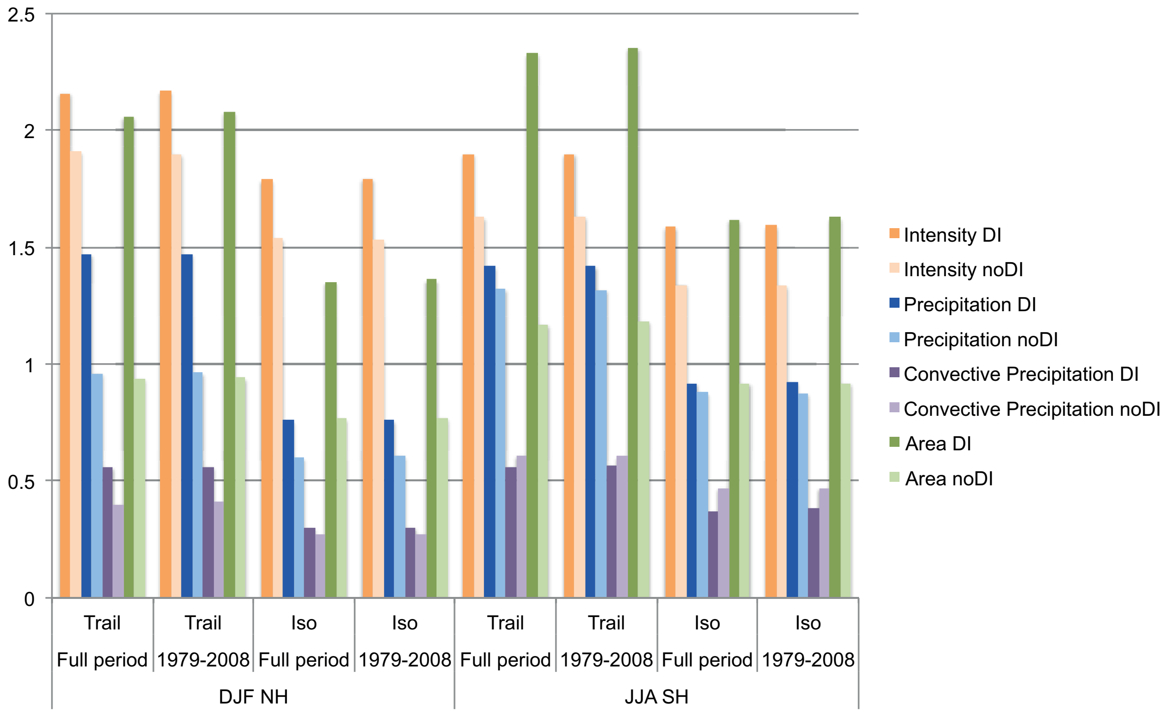

Figure S1: Bar chart comparing the front characteristics for the full period (1979–2014) and the shorter period (1979–2008; when ERA-Interim sea surface temperature resolution is low). Front intensity (K/100km), average precipitation (mm/6hr), convective precipitation (mm/6hr), and front area (km^2 $\times$ 10^6).

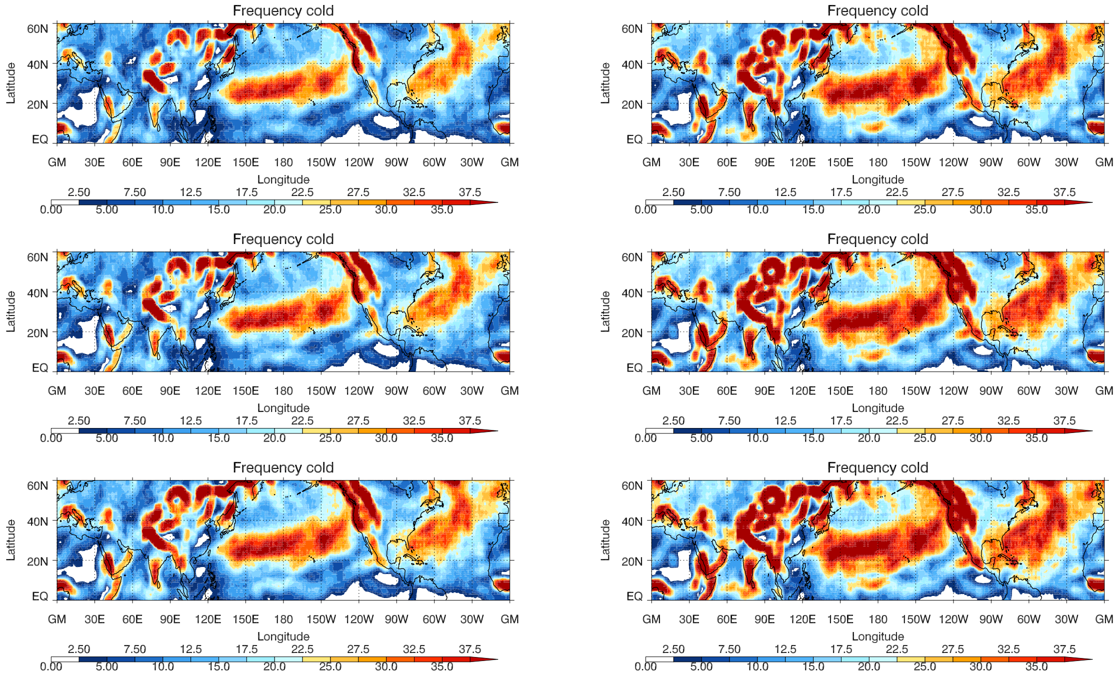

Figure S2: Sensitivity to front identification parameters. Frequency of all cold fronts (% of all times) for 2005 January and February. Left column shows with a minimum length requirement of 5 grid points, and right column no minimum length requirement, for search radius of (top) 2 degrees, (middle) 3 degrees, and (bottom) 4 degrees. The left middle panel shows the results for the parameters used in the paper.

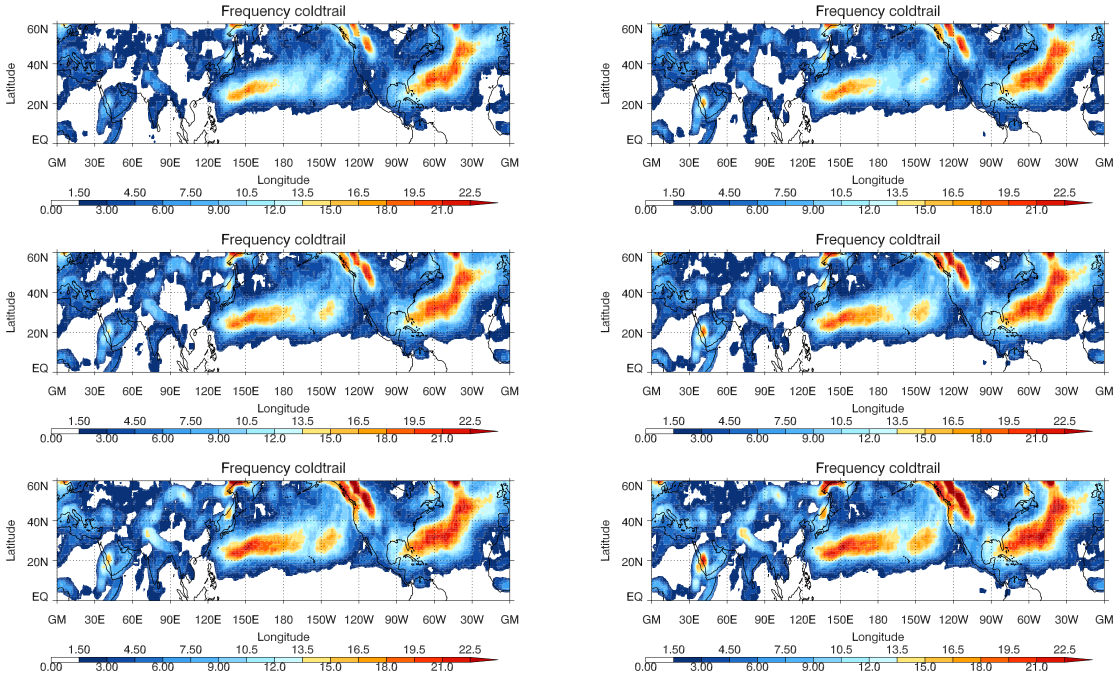

Figure S3: As Figure S2 but for trailing fronts.

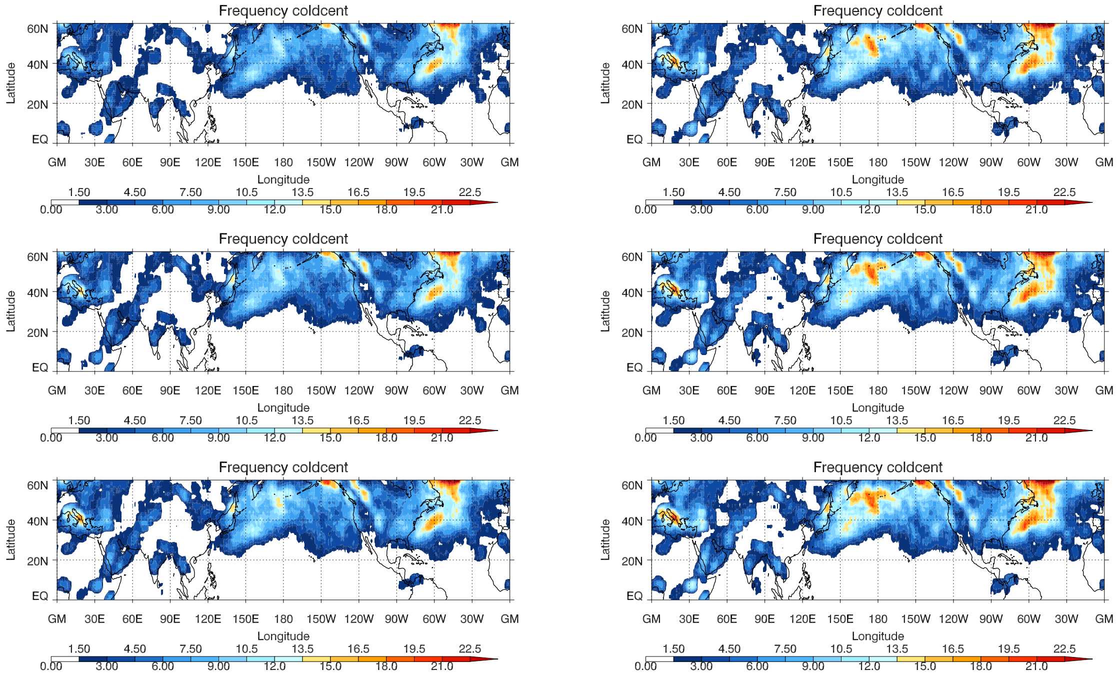

Figure S4: As Figure S2 but for central fronts.

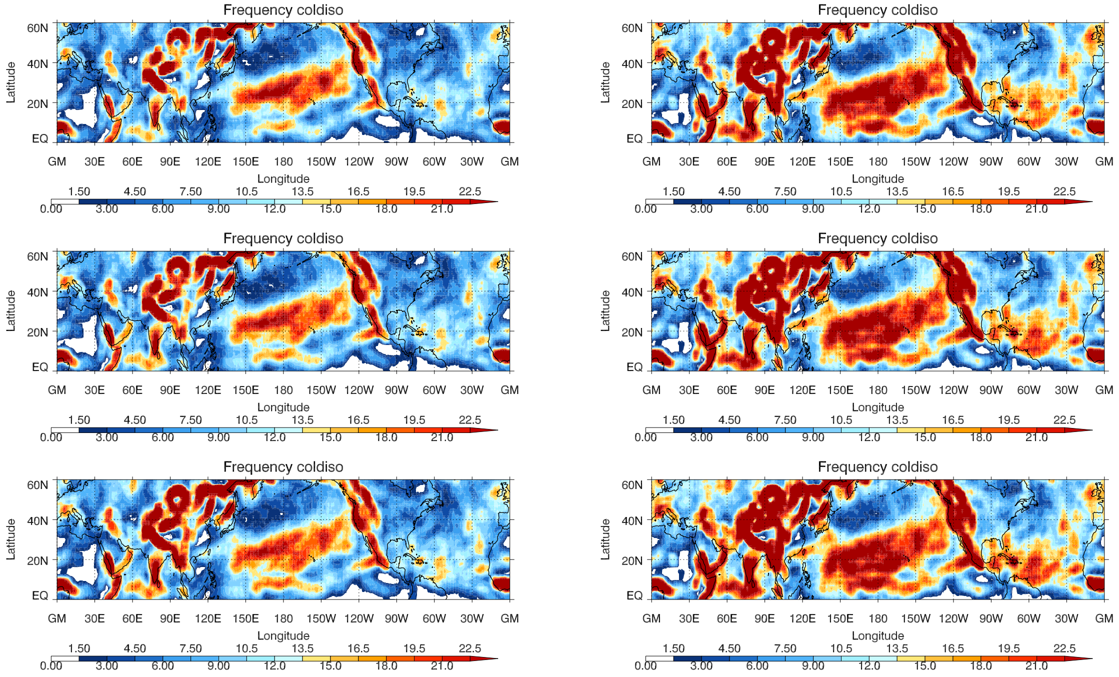

Figure S5: As Figure S2 but for isolated fronts.

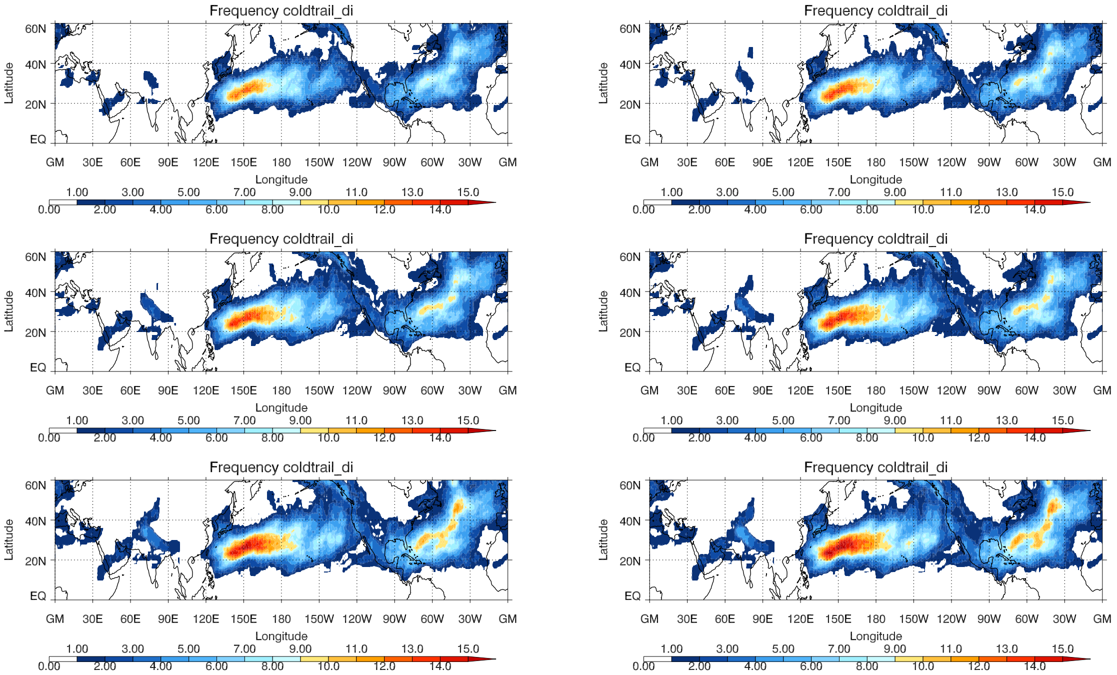

Figure S6: As Figure S2 but for trailing fronts associated with DIs.

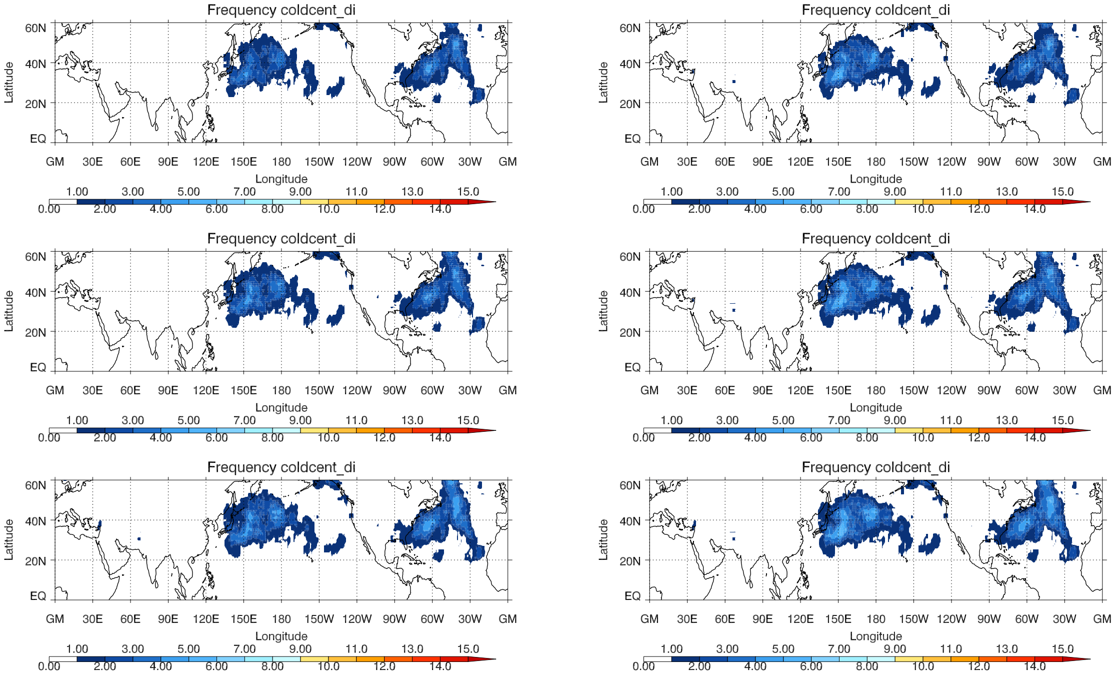

Figure S7: As Figure S2 but for central fronts associated with DIs.

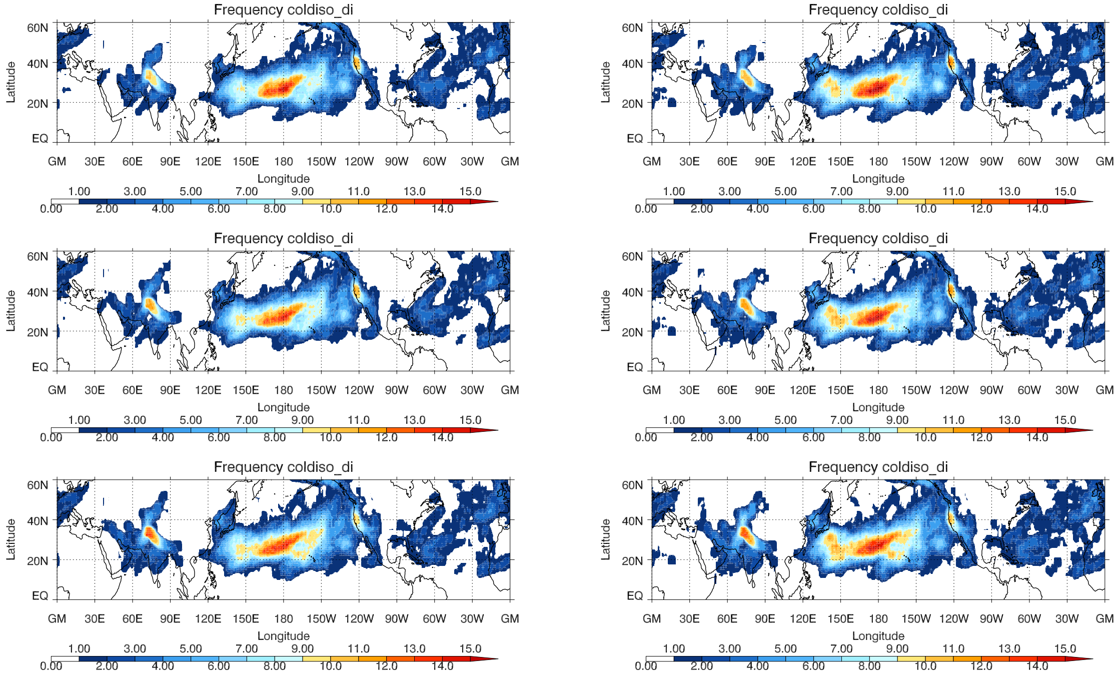

Figure S8: As Figure S2 but for isolated fronts associated with DIs.

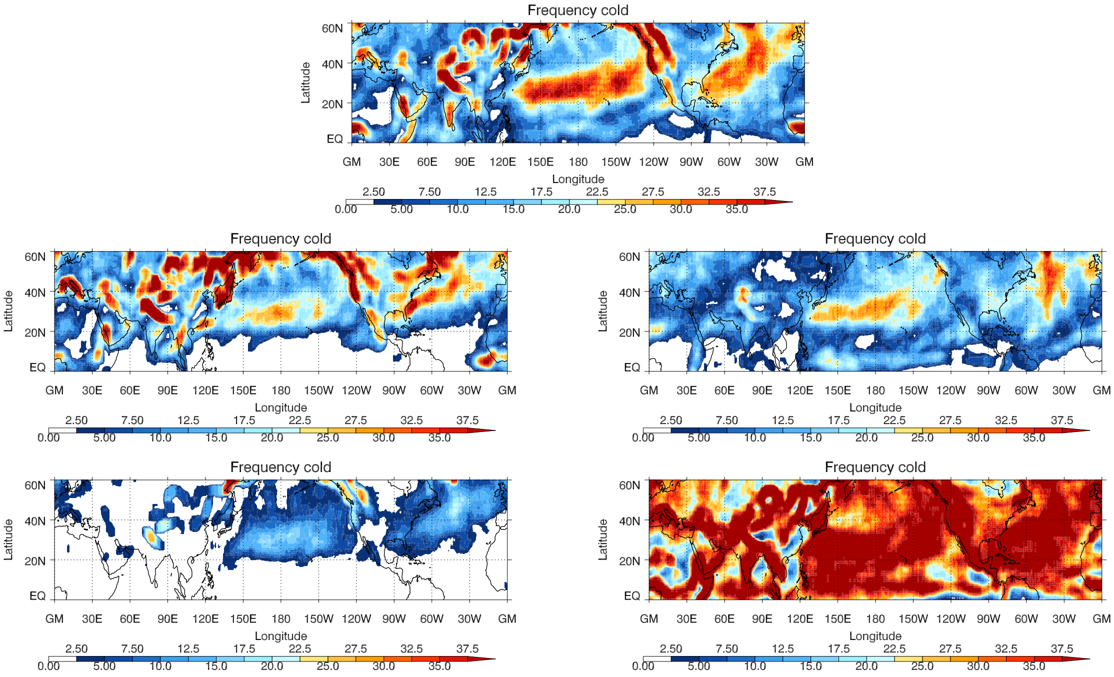

Figure S9: Sensitivity to level and threshold. Frequency of all cold fronts (% of all times) for 2005 January and February. The top panel shows the results for the parameters used in the paper (850 hPa, 3 degree search radius, minimum length of 5 grid points). The middle row shows the results identifying fronts on 925hPa on the left and 700hPa on the right. The bottom row shows results for a doubled thermal front parameter threshold on the left, and a halved thermal front parameter threshold on the right.

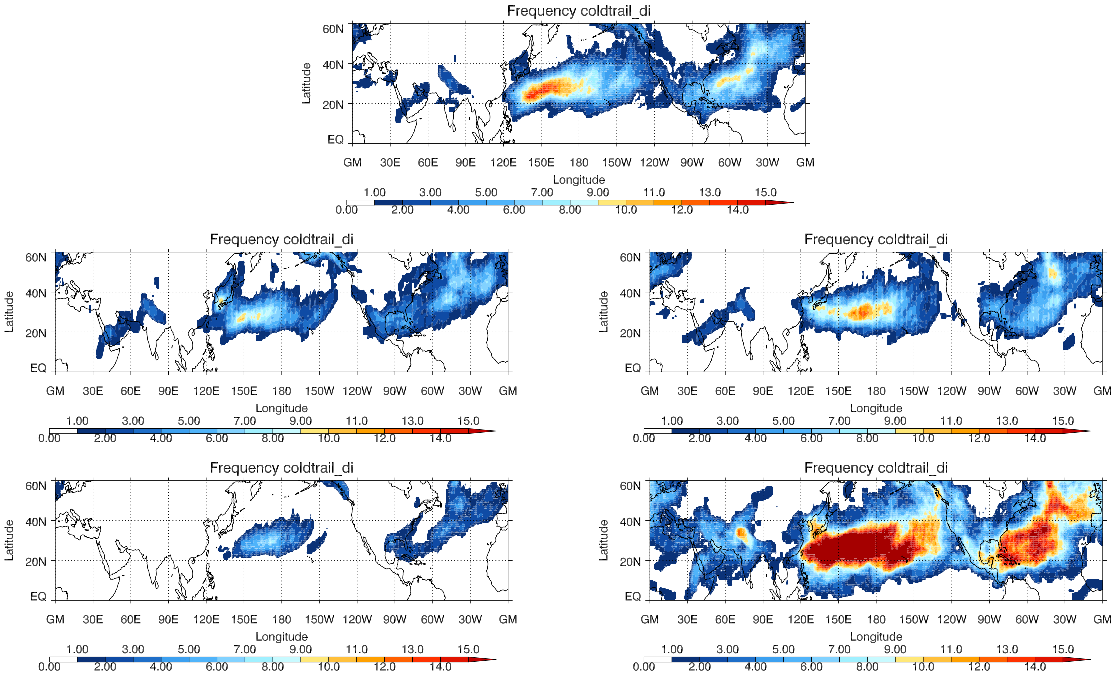

Figure S10: As S9 but for trailing fronts associated with DIs.

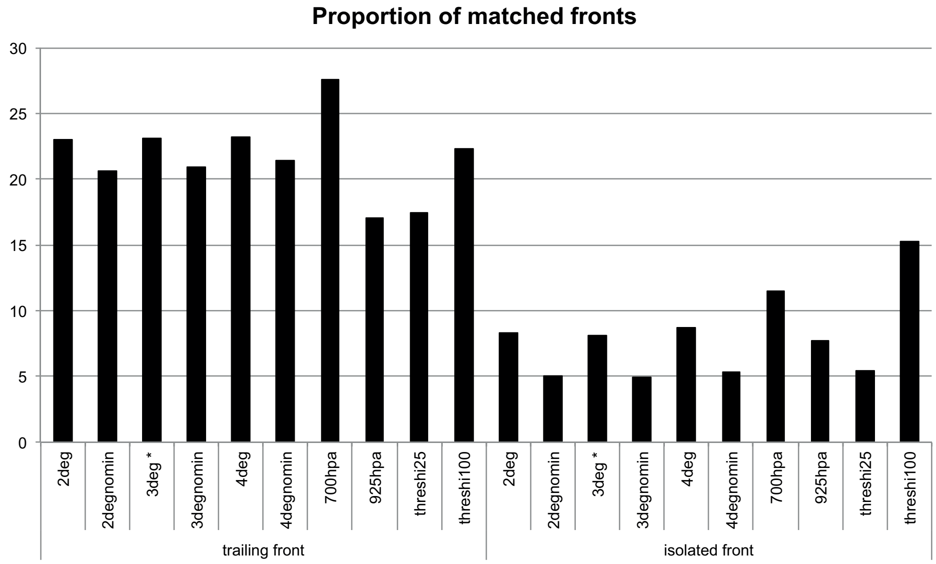

Figure S11: Proportion of fronts matched with dry intrusions for different front identification parameters and levels for January and February 2005. 3*deg* refers to the search radius in the line-joining algorithm, *nomin* refers to the requirement for a minimum length of 5 grid points being removed. The levels of 700 hPa and 925 hPa are shown by 700*hpa* and 925*hpa* respectively, the doubled threshold shown by *threshi*100, and the halved threshold shown by *threshi*25. The set marked with an asterix corresponds to the method used in the rest of this study (850 hPa, 3 degrees search radius, and minimum length requirement).

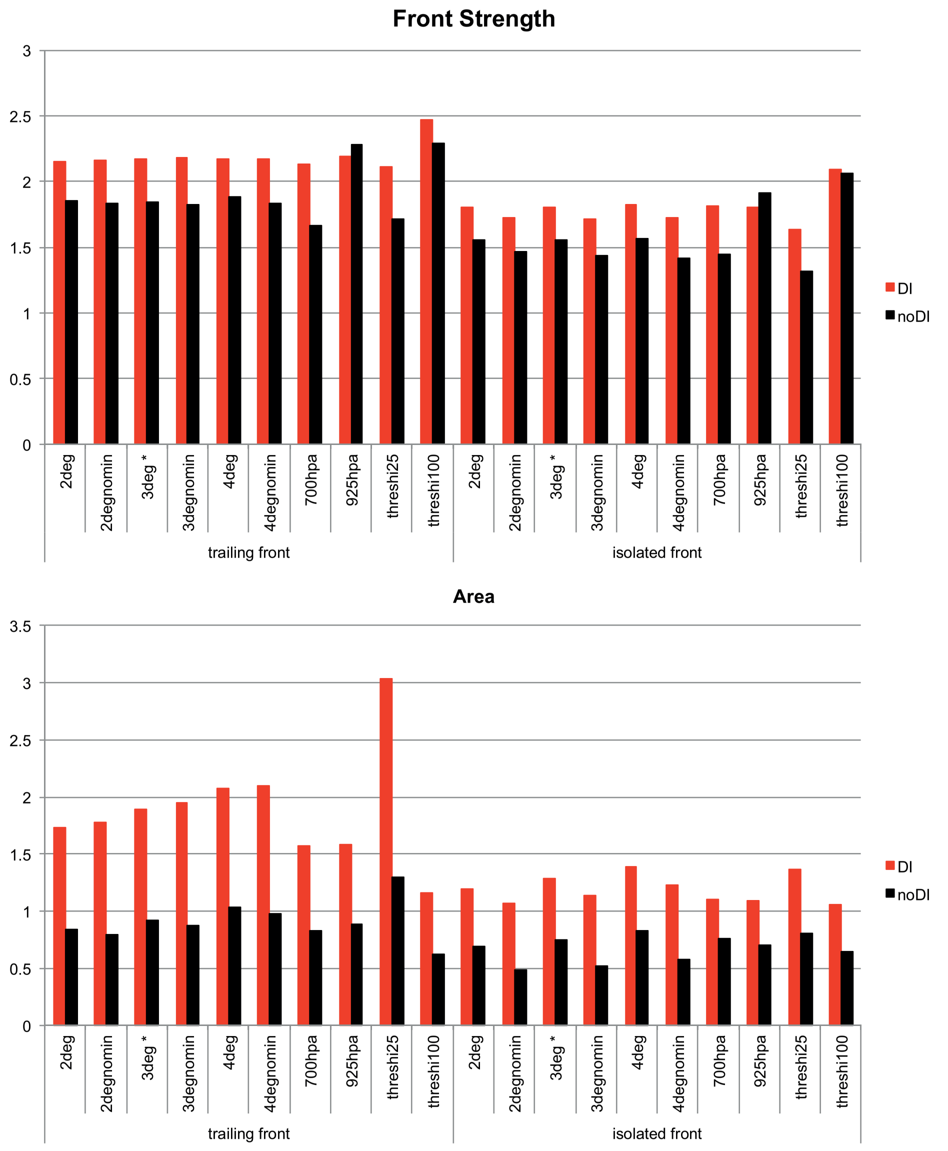

Figure S12: Mean characteristics of the fronts for different front identification parameters and levels for January and February 2005. Top - front strength, bottom, front area. See Figure S11 for explanation of labels.

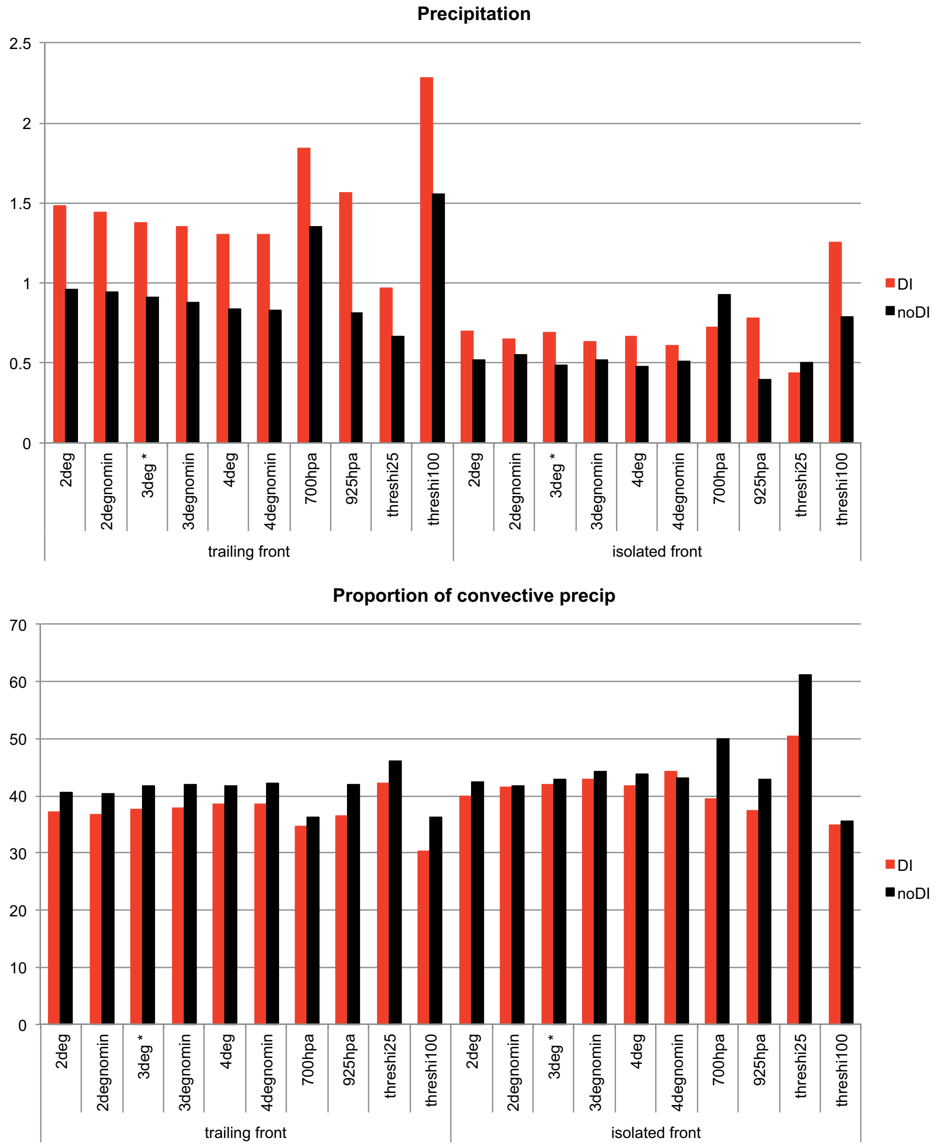

Figure S13: Mean characteristics of the fronts for different front identification parameters and levels for January and February 2005. Top - precipitation, bottom - proportion of precipitation that is convective. See Figure S11 for explanation of labels.
